# Metabolic Stress‐Induced Choline Kinase α (CHKA) Activation in Endothelial Subpopulation Contributes to Diabetes‐Associated Microvascular Dysfunction

**DOI:** 10.1002/advs.202417045

**Published:** 2025-06-23

**Authors:** Ling Ren, Linyu Zhang, Yun Bai, Chang Huang, Xiaosa Li, Fanfei Ma, Wan Mu, Mudi Yao, Chang Jiang, Xiangjun Chen, Qin Jiang, Biao Yan

**Affiliations:** ^1^ Department of Ophthalmology, Shanghai General Hospital, School of Medicine Shanghai Jiao Tong University Shanghai 200080 China; ^2^ Eye Institute and Department of Ophthalmology, Eye & ENT Hospital, Shanghai Medical College Fudan University Shanghai 200030 China; ^3^ The Affiliated Eye Hospital Nanjing Medical University Nanjing 210000 China; ^4^ College of Information Science Shanghai Ocean University Shanghai 201306 China; ^5^ Eye Center of the Second Affiliated Hospital Zhejiang University School of Medicine Hangzhou 310009 China; ^6^ Institute of Translational Medicine Zhejiang University School of Medicine Hangzhou 310020 China

**Keywords:** diabetic retinopathy, endothelial cells, heterogeneity, single cell RNA sequencing

## Abstract

Diabetes is a prevalent metabolic disorder, and its associated microvascular dysfunction plays a key role in the pathogenesis of complications such as retinopathy, nephropathy, and peripheral vascular disease. However, the mechanism governing metabolic regulation and functional heterogeneity of endothelial dysfunction remains incompletely understood. This study investigates the role of metabolic stress‐induced activation of choline kinase α (CHKA) in endothelial cell (EC) subpopulations, contributing to diabetes‐induced microvascular dysfunction. Using single‐cell RNA sequencing (scRNA‐seq), three distinct EC subclusters are identified within retinal vessels. Among them, one subcluster characterized by elevated CHKA expression is associated with enhanced angiogenic activity. CHKA silencing in ECs inhibited angiogenic effects and reduced retinal vascular dysfunction in diabetic murine models. CHKA silencing also disrupted NAD^+^ metabolism, causing reduced NAD^+^ levels. Supplementation with nicotinamide mononucleotide (NMN), a precursor of NAD^+^, partially reversed the anti‐angiogenic effects induced by CHKA silencing. Mechanistically, CHKA regulated endothelial dysfunction through the NAD^+^‐SIRT1‐Notch signaling. Clinical sample analysis and Mendelian randomization studies provided strong evidence linking increased CHKA expression with diabetic microvascular complications. Collectively, this study advances the understanding of endothelial heterogeneity and identifies CHKA as a critical regulator of pathological angiogenesis, highlighting its potential as a therapeutic target for diabetic vascular complications.

## Graphical abstract

This study uncovers CHKA as a pivotal driver of vascular dysfunction in diabetic retinopathy and highlights its role in endothelial dysfunction through NAD⁺‐SIRT1‐Notch signaling. Single‐cell and functional analyses, combined with clinical and genetic validation, suggest CHKA as a promising therapeutic target for the prevention and treatment of diabetic microvascular complications.

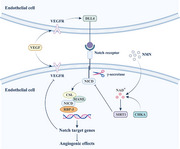

## 1 Introduction

Diabetes mellitus is becoming a global health crisis, which will affect ≈700 million individuals worldwide by 2045.^[^ ^1^ ^]^ This metabolic disorder not only gives rise to systemic complications but also disrupts microvascular integrity, thereby leading to debilitating conditions such as blindness, renal failure, cardiovascular disease, and peripheral neuropathy.^[^ ^2^, ^3^, ^4^ ^]^ Diabetes‐associated microvascular complications pose a substantial burden on global healthcare systems. Despite the availability of treatments such as anti‐Vascular endothelial growth factor (VEGF) therapy, their clinical efficacy remains limited. Diabetic retinopathy (DR) is a key model for studying diabetes‐induced microvascular complications. It is characterized by vascular leakage and pathological angiogenesis, which can lead to severe complications such as hemorrhage and retinal detachment.^[^ ^5^, ^6^, ^7^ ^]^ Similar microvascular damage also affects the kidneys, heart, and peripheral tissues, contributing to the development of nephropathy, myocardial ischemia, and neuropathy. Although these complications are organ‐specific, they share common pathological features, including endothelial dysfunction, vascular inflammation, and localized vascular occlusion.^[^ ^8^, ^9^, ^10^ ^]^ Thus, understanding the vascular changes underlying DR not only advances our understanding within the field of ophthalmology but also offers insights into the broader mechanism of diabetes‐related vascular dysfunction across multiple organ systems.

Endothelial cell (EC) dysfunction, an early indicator of diabetes‐related vascular disease, drives pathological changes across multiple organs.^[^ ^11^, ^12^ ^]^ As vital components of the vascular network, ECs play a crucial role in maintaining vascular stability and regulating pathological angiogenesis.^[^ ^13^, ^14^ ^]^ Emerging studies have highlighted endothelial functional diversity across different tissues and organs. For example, glomerular ECs in the kidney specialize in filtration to regulate fluid metabolism, while lung microvascular ECs facilitate gas exchange for oxygenation.^[^ ^15^, ^16^ ^]^ In the liver, sinusoidal ECs support nutrient exchange with hepatocytes, playing a key role in metabolic homeostasis.^[^ ^17^ ^]^ Brain ECs contribute to blood‐brain barrier, regulating molecular passage to maintain neural stability and protect against neurotoxic agents.^[^ ^18^, ^19^ ^]^ In the retina, ECs also display functional diversity: “tip cells” guide new vessel growth through active migration and environmental sensing, while “stalk cells” contribute to vessel extension and stability.^[^ ^20^, ^21^ ^]^ Additionally, ECs in static vessels primarily maintain barrier integrity, whereas those in dynamic vessels are important for vascular remodeling. The functional heterogeneity offers valuable insights into their roles in vascular‐related conditions. Thus, investigating EC heterogeneity in diabetic environments is essential for clarifying the mechanisms underlying diabetes‐related microvascular diseases.

Clarifying EC heterogeneity has opened new avenues for precision therapies targeting specific EC subpopulations across distinct diseases. Distinct EC subsets play unique roles in disease progression, presenting opportunities for tailored interventions. For instance, unique EC subgroups identified within glioblastoma may enhance targeted drug delivery in central nervous system malignancies.^[^ ^22^ ^]^ Similarly, the CD133^+^TRPV4^high^ EC subpopulation in coronary arteries, which is linked to vascular relaxation and blood flow regulation, presents potential targets for interventions in vascular injury.^[^ ^23^ ^]^ However, the specific roles of EC subpopulations in diabetes‐related microvascular disease are not yet fully understood. Single‐cell RNA sequencing (scRNA‐seq) is a powerful tool for exploring cellular heterogeneity, allowing for the identification of unique functional markers and characteristics within distinct EC subpopulations.^[^ ^24^, ^25^ ^]^ Exploring endothelial heterogeneity by scRNA‐seq may provide valuable insights for developing more precise and personalized therapeutic strategies to target diabetic microvascular complications.

In this study, we constructed a comprehensive single‐cell atlas of healthy and diabetic murine retinas to investigate endothelial heterogeneity. We identified three distinct EC subclusters, one of which was closely associated with retinal vascular dysfunction. Choline kinase alpha (CHKA), a key enzyme in choline phospholipid metabolism, was predominantly expressed in this EC subcluster and significantly up‐regulated in DR. CHKA silencing exerted anti‐angiogenic effects in vitro and alleviated retinal vascular dysfunction through the NAD^+^‐SIRT1‐Notch signaling axis in vivo. Taken together, this study not only advances the understanding of EC heterogeneity in diabetic vascular complications but also positions CHKA as a promising target for intervention in these diseases.

## 2 Results

### 2.1 Single‐Cell Profiling Identifies Distinct Endothelial Cell Subclusters and their Functional Characteristics

**Figure 1:**
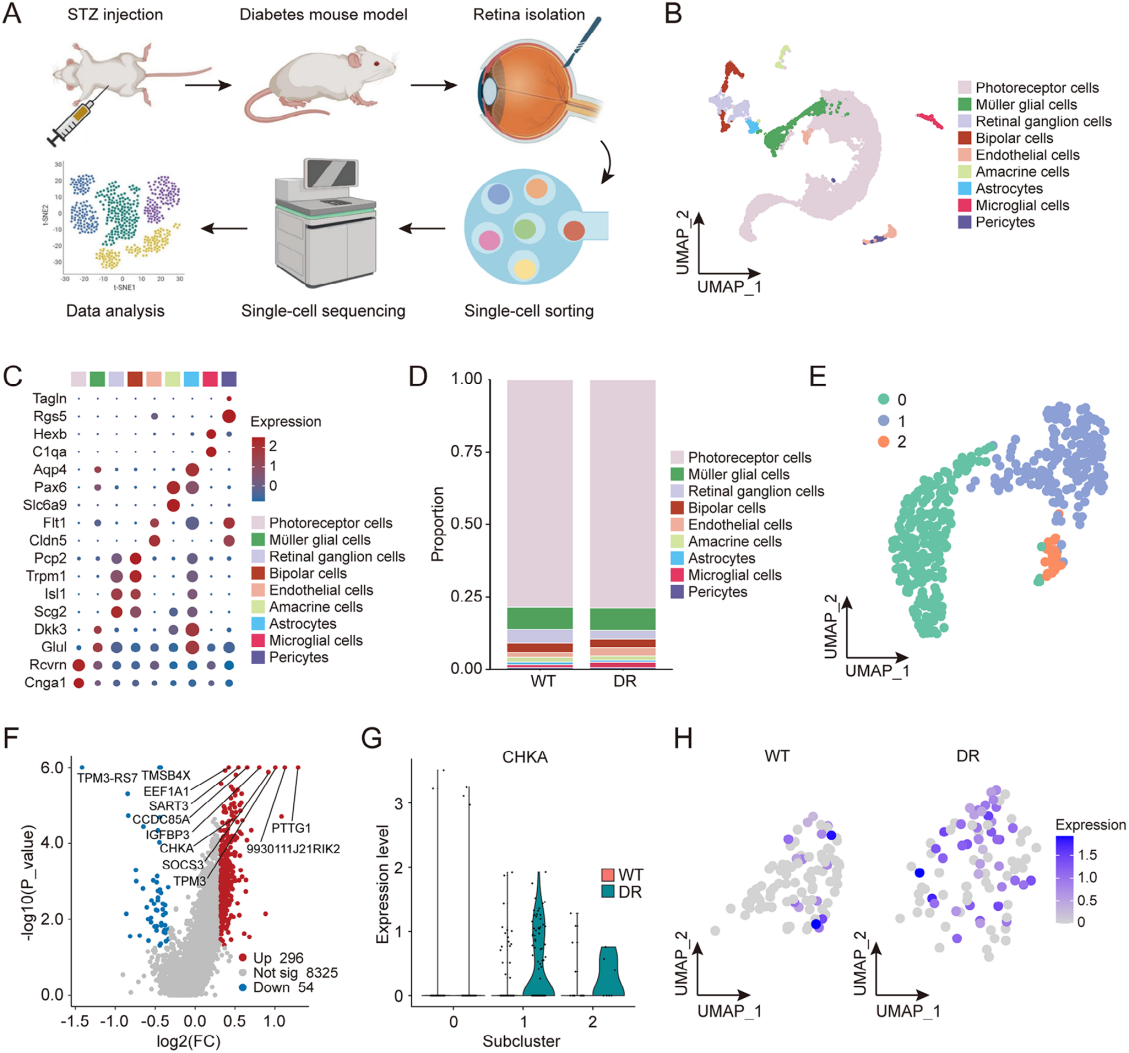
Single‐cell profiling identifies distinct endothelial cell subclusters and their functional characteristics. A) The flowchart describes the procedure for creating a single‐cell transcriptome atlas. Diabetes retinopathy was built through STZ injection, and after a duration of 25 weeks, the mice were sacrificed to isolate the retinas and generate single‐cell suspensions. Following this, sequencing libraries were prepared and subsequently analyzed. B) UMAP plot displays the identification of various cell types. Each color corresponds to a specific cell type, such as photoreceptor cells, Müller glial cells, astrocytes, retinal ganglion cells, bipolar cells, endothelial cells, amacrine cells, microglial cells and pericytes. C) Dot plot displays the average expression levels of selected markers specific to different cell types, which validate each identified cell cluster. D) The barplot depicts the proportion of various retinal cell types in non‐diabetic (WT) and diabetic (DR) group. E) UMAP plot reveals the identification of three distinct subclusters of endothelial cells. F) Volcano plot displays the differentially expressed genes between diabetic and non‐diabetic groups within subcluster 1. G) Violin plot shows the expression levels of CHKA gene across three endothelial cell subclusters. H) Feature plots illustrate the expression levels of CHKA gene for both WT and DR group within subcluster 1.

To investigate endothelial cell heterogeneity and its contribution to DR pathogenesis, we generated a diabetic murine model using streptozotocin (STZ) induction, which can recapitulate hyperglycemia‐induced retinal vasculopathy observed in human DR. Upon validation of the model, retinal tissues were harvested from both diabetic and non‐diabetic control mice. The retinas were dissociated to generate high‐viability single‐cell suspensions (viability >85%) for subsequent scRNA‐seq analysis (**Figure** **1**A).

Following obtaining the sequencing data, we performed quality filtering and batch correction. We established the criteria for filtering, including the number of genes identified in each cell (nFeature_RNA), the total RNA count across all genes in each cell (nCount_RNA), and the proportion of mitochondrial genes relative to total genes in each cell (percent.mt), all of which are detailed in Figure S1A (Supporting Information). The correlation analysis revealed a strong relationship between nFeature_RNA and nCount_RNA, with a correlation coefficient of 0.90 observed for both non‐diabetic group and diabetic group (Figure S1B, Supporting Information). Following quality control, we obtained a total of 19648 cells from non‐diabetic group and 20926 cells from diabetic group. These cells were subsequently categorized into 38 transcriptionally distinct clusters, visually represented in 2D space using the Uniform manifold approximation and projection (UMAP) algorithm (Figure S1C, Supporting Information).

For cell type annotation, we analyzed the average expression profiles of canonical marker genes within each cluster, which enabled the classification of nine distinct retinal cell types (Figure 1B), including photoreceptor cells, Müller glial cells, retinal ganglion cells, bipolar cells, endothelial cells, amacrine cells, astrocytes, microglial cells, and pericytes. The spatial distribution of these cell types within the retinal microenvironment was visualized using 3D UMAP (Figure S1E, Supporting Information). The average expression levels of these specific markers validated the identified clusters (Figure 1C). Comparative cellular composition analysis revealed significant differences in cell type proportions between diabetic and non‐diabetic retinas (Figure 1D).

UMAP analysis of retinal endothelial cells identified 3 transcriptionally distinct subclusters (Figure 1E; Figure S1F, Supporting Information). Representative genes with high expression in each subcluster are shown in Figure S1D (Supporting Information). Subcluster 0 was enriched for genes involved in extracellular matrix binding, energy metabolism, translation, RNA and actin binding, vascular regulation, and blood pressure control. Subcluster 1 showed high expression of angiogenesis‐ and remodeling‐related genes including Cxcl12, Flt1, Ptprb, Klf4, and Spock2. Subcluster 2 expressed genes linked to ion transport, protein methylation, cytoskeletal organization, and membrane potential regulation.

To further explore subcluster 1, we performed Gene Ontology (GO) enrichment analysis on the top 300 most highly expressed genes in this subcluster across GO‐Biological Process (GO‐BP), GO‐Cellular Component (GO‐CC), and GO‐Molecular Function (GO‐MF) (Figure S1G–I, Supporting Information). GO‐BP terms such as “Angiogenesis”, “Blood vessel morphogenesis”, “MAPK cascade regulation”, and “VEGF response” were significantly enriched. GO‐CC analysis indicated localization to cell surface, cytoskeleton, cell junctions, and cytoplasm, while GO‐MF terms included “Actin binding”, “Integrin binding”, “Cadherin binding”, and “VEGF receptor activity”. KEGG and REACTOME pathway enrichment further supported involvement in cell adhesion, proliferation, and VEGF signaling (Figure S1J, Supporting Information), implying subcluster 1′s association with pathological angiogenesis.

To assess diabetic‐specific changes, we compared gene expression in subcluster 1 between diabetic and control groups (Figure 1F). CHKA was significantly up‐regulated in diabetic group (FC = 1.5, *P* value = 3.3×10⁻⁵) and predominantly expressed in subcluster 1 (Figure 1G). FeaturePlot analysis revealed a marked increase in CHKA⁺ cells in the DR group (46%) compared to controls (18%) (Figure 1H). Given subcluster 1′s role in pathological angiogenesis, these results suggest that CHKA may contribute to angiogenic processes in DR. To further demonstrate cross‐species consistency, we analyzed a publicly available scRNA‐seq dataset (GEO: GSE209872) of diabetic rat retinas (2, 4, and 8 weeks) and observed a similarly significant up‐regulation of CHKA in retinal ECs of diabetic rats (Figure S2, Supporting Information), underscoring the robustness of our findings.

### 2.2 CHKA Expression is Elevated and Primarily Localized to ECs

**Figure 2:**
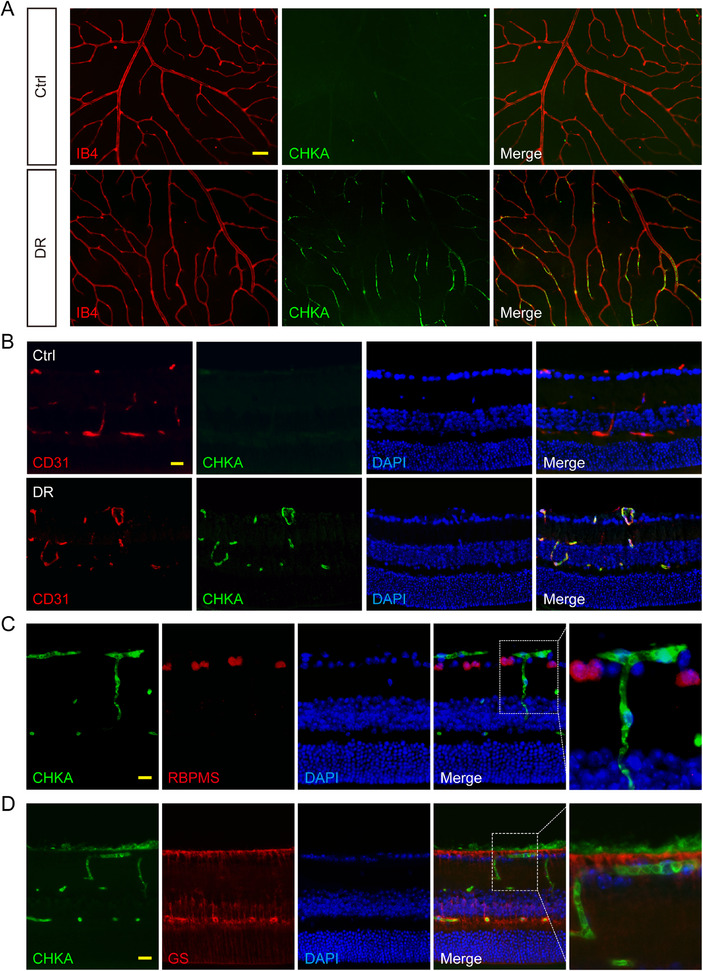
CHKA expression is elevated and primarily localized to endothelial cells. A) Representative immunofluorescence images of retinal flat mounts demonstrating the expression of CHKA in non‐diabetic (Ctrl) and diabetic mouse retinas (DR). IB4 staining was used to identify retinal vessels. Scale bar: 20 µm. B) Immunofluorescence analysis of frozen retinal sections revealing the co‐localization of CHKA with CD31. CD31 was used to mark endothelial cells. DAPI was used to visualize cell nuclei. Scale bar: 20 µm. C,D) Immunofluorescence assays were conducted to detect the co‐localization of CHKA with RBPMS (marking retinal ganglion cells, C) and GS (identifying astrocytes and Müller glial cells, D). Scale bar: 20 µm.

Based on the results of scRNA‐seq, we performed immunofluorescence staining to detect CHKA expression pattern in retinal tissues. CHKA expression was significantly elevated in the retinas of diabetic mice compared to non‐diabetic controls, with clear co‐localization with the vascular marker Isolectin B4 (IB4), as observed in retinal flat mounts (**Figure** **2**A). Further validation by frozen retinal sections confirmed CHKA enrichment in CD31‐positive ECs and showed increased CHKA levels in diabetic group (Figure 2B), implicating CHKA in endothelial pathology under diabetic condition. In contrast, CHKA expression did not co‐localize with retinal ganglion cell marker RBPMS or glial markers such as glutamine synthetase (GS), which labels Müller glia and astrocytes (Figure 2C,D), indicating that CHKA up‐regulation is largely restricted to retinal ECs during DR progression.

To further explore the role of CHKA in pathological angiogenesis, we examined retinal flat mounts from oxygen‐induced retinopathy (OIR) model. CHKA was prominently localized to neovascular tufts in OIR retinas, demonstrating its association with aberrant angiogenic regions (Figure S3, Supporting Information). Collectively, these findings highlight the endothelial‐specific up‐regulation of CHKA and suggest its potential involvement in endothelial dysfunction and pathological angiogenesis.

### 2.3 Silencing of CHKA Suppresses Endothelial Angiogenic Effects In Vitro

**Figure 3:**
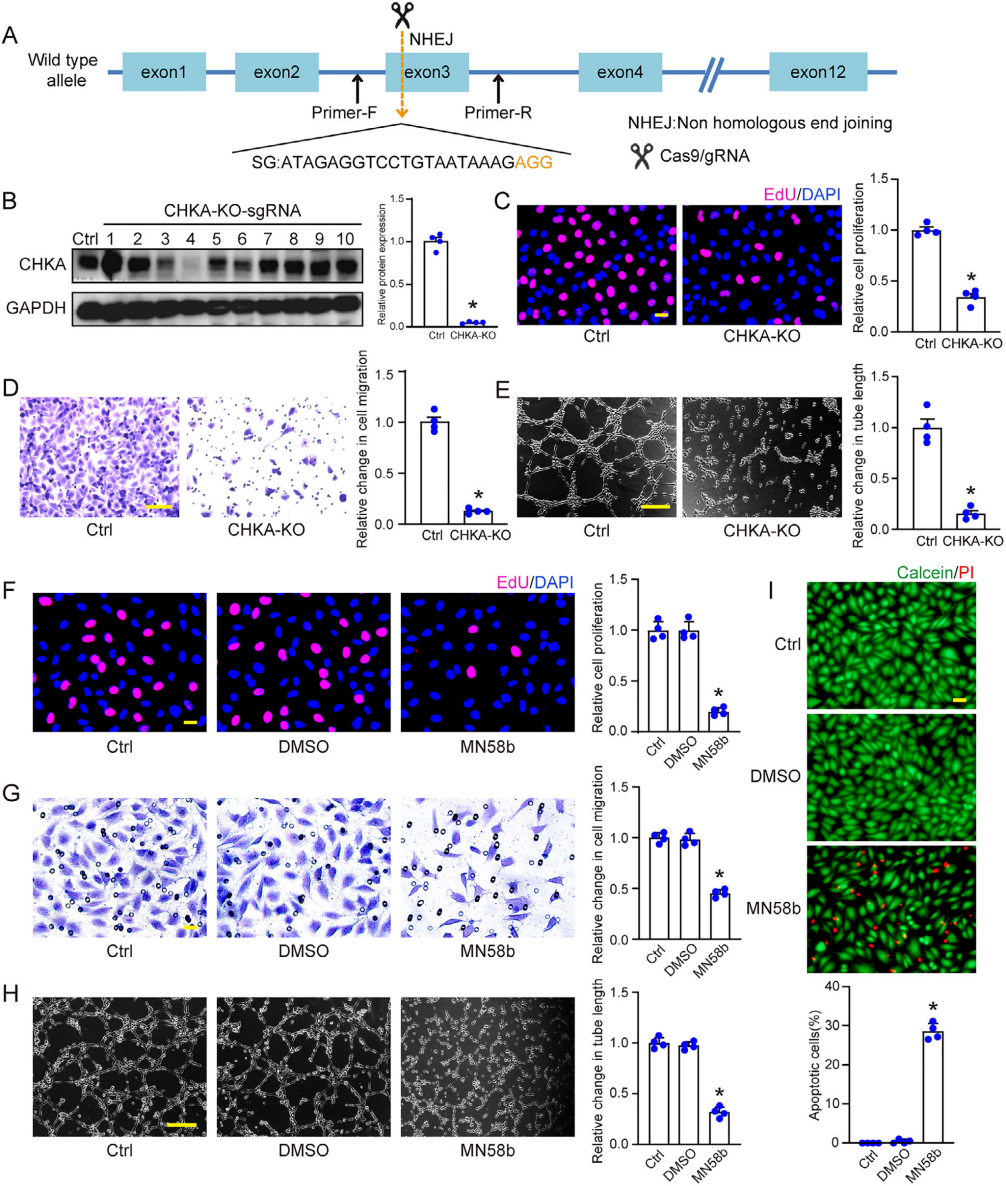
Knockout and inhibition of CHKA suppresses endothelial cell angiogenic effects in vitro. A) Schematic illustration of CRISPR/Cas9‐mediated CHKA gene knockout in HRMECs. The guide RNA (gRNA) targets exon 3 of CHKA gene, inducing a double‐strand break (DSB) at the specific site marked by the scissors. The DSB is repaired by non‐homologous end joining (NHEJ), leading to the introduction of frameshift mutations and subsequent gene knockout. The sequence of the single guide RNA (sgRNA) is indicated below, with the protospacer adjacent motif (PAM) sequence shown in orange. B) Western blot analysis of CHKA expression in HRMECs transfected with CHKA‐KO‐sgRNA, showing CHKA protein levels in control (Ctrl) and single‐cell clones 1–10. Clone 4 was identified as successfully knocked out (CHKA‐KO). GAPDH was detected as the loading control. Densitometric analyses show relative protein expression of CHKA in Ctrl and CHKA‐KO cells. n = 4; *P < 0.05; Student's *t* test. C) Cell proliferation of CHKA‐KO HRMECs was detected using EdU staining assay. HRMECs were seeded in 24‐well plates at a density of 5 × 10⁴ cells per well, followed by EdU incubation for 3 h. Scale bar: 20 µm. D) Transwell assay was conducted to detect cell migration ability. Scale bar: 50 µm. E) Tube formation assay was conducted to detect tube formation ability. Scale bar: 100 µm. n = 4; *P < 0.05; Student's *t* test. F‐I) HRMECs were treated with CHKA protein inhibitor MN58b (10 µm), DMSO (solvent control), or left untreated (Ctrl) for 24 h, then these cells were subjected to EdU staining assay (F, scale bar: 20 µm), transwell assay (G, scale bar: 20 µm), and tube formation assay (H, scale bar: 100 µm). I) Calcein‐AM/PI staining was conducted for the assessment of live (green) and apoptotic (red) cells. Scale bar: 20 µm; n = 4; *P < 0.05; One‐way ANOVA with Bonferroni post hoc test.

Based on the above‐mentioned analysis, CHKA emerges as a key regulator of endothelial angiogenic effects. To explore its role, we generated a CHKA knockout (CHKA‐KO) model in human retinal microvascular endothelial cells (HRMECs) using CRISPR/Cas9‐mediated genome editing. Successful knockout of CHKA was confirmed by western blot analysis (**Figure** **3**A,B). CHKA‐KO HRMECs exhibited a significantly reduced number of EdU‐positive cells compared to the control group, indicating a marked reduction of cell proliferation (Figure 3C). Transwell migration assays revealed a marked reduction in the migratory capacity of CHKA‐KO cells compared to the controls (Figure 3D). Moreover, tube formation assays demonstrated that CHKA knockout impaired the ability of HRMECs to form capillary‐like structures on Matrigels (Figure 3E). Consistent results were observed with CHKA silencing using small interfering RNAs (siRNAs), which also led to decreased cell viability, proliferation, migration, and tube formation (Figure S4, Supporting Information). In contrast, overexpression of CHKA enhanced these endothelial effects in vitro (Figure S5, Supporting Information), further supporting the pro‐angiogenic role of CHKA.

To evaluate the therapeutic potential of pharmacological inhibition, we treated HRMECs with MN58b, a selective CHKA inhibitor.^[^ ^26^ ^]^ Treatment with MN58b suppressed endothelial proliferation, migration, and tube formation compared to DMSO group and untreated controls (Figure 3F–H). This concentration was selected based on prior study demonstrating its efficacy in inhibiting CHKA activity in vitro.^[^ ^27^ ^]^ In addition, MN58b treatment induced endothelial apoptosis, as indicated by a marked increase in PI‐positive cells (Figure 3I).

### 2.4 Intervention Targeting CHKA Activity Suppresses Pathological Angiogenesis In Vivo

**Figure 4:**
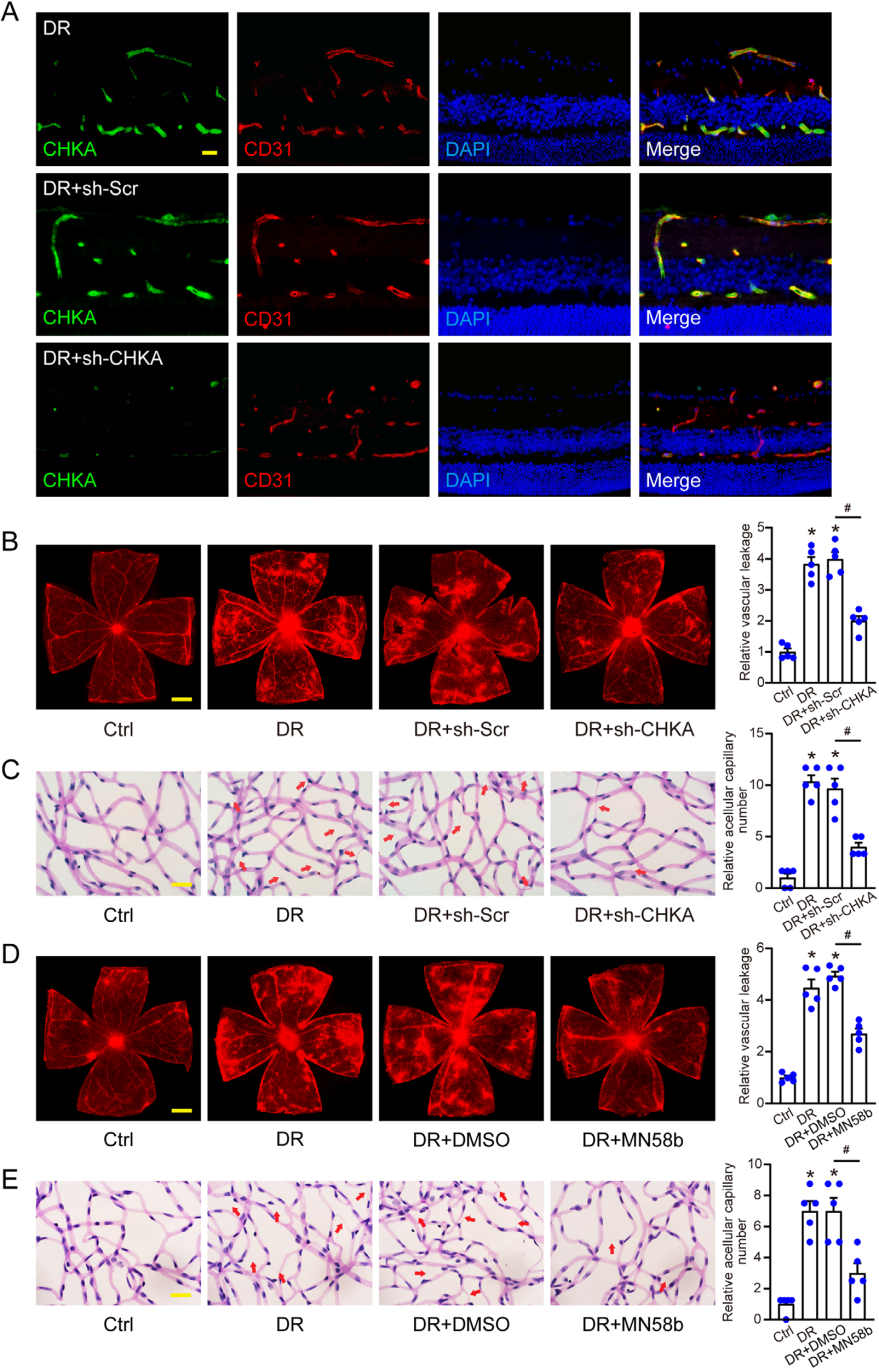
Intervention targeting CHKA activity suppresses pathological angiogenesis in vivo. A) Immunofluorescence staining of frozen retinal sections was conducted to assess the efficacy of CHKA knockdown. Following intravitreal injection of sh‐Scr AAV, sh‐CHKA AAV, or no treatment (DR), the mice were analyzed after 4 weeks to evaluate CHKA expression levels in endothelial cells. Scale bar: 20 µm. B,C) DR mice received intravitreal injections of sh‐Scr AAV, sh‐CHKA AAV, or left untreated (DR), with age‐matched non‐diabetic mice taken as controls (Ctrl). Evans blue assays (B) assessed retinal vascular leakage. Scale bar: 500 µm. Trypsin digestion followed by PAS staining (C) identified acellular capillaries in the retina. Acellular capillaries were labelled using red arrows. Scale bar: 10 µm. D,E) DR mice received weekly intravitreal injections of vehicle control (0.25% DMSO) or MN58b (50 µm) for 2 weeks, while age‐matched non‐diabetic mice acted as controls (Ctrl). Evans blue assays (D, Scale bar: 500 µm) and trypsin digestion with PAS staining (E, Scale bar: 10 µm) were conducted to evaluate retinal vascular leakage and acellular capillaries. n = 5; *P < 0.05 compared to Ctrl group; ^#^ *P* < 0.05; One‐way ANOVA with Bonferroni post hoc test.

To further investigate the role of CHKA in vivo, we employed a diabetic murine model to evaluate the effects of CHKA inhibition on retinal vascular dysfunction. Adeno‐associated virus (AAV) vectors with a specific promoter targeting endothelial cells (sh‐CHKA AAV) were employed to selectively reduce CHKA expression in retinal endothelial cells. The mice were intravitreally injected with sh‐CHKA AAV, a control vector carrying a scrambled shRNA (sh‐Scr AAV), or left untreated. After 4 weeks, CHKA expression levels in retinal endothelial cells were assessed by immunofluorescence staining. The sh‐CHKA AAV group exhibited a significant reduction in CHKA fluorescence intensity compared to both the sh‐Scr AAV and untreated DR groups, confirming efficient knockdown of CHKA in endothelial cells (**Figure** **4**A). Evans blue assays were conducted to detect retinal vascular permeability. As shown in Figure 4B, diabetic mice demonstrated significant retinal vascular leakage compared to age‐matched controls. However, intravitreal injection of sh‐CHKA AAV led to a notable reduction in vascular leakage. Trypsin digestion followed by periodic acid‐Schiff (PAS) staining revealed that CHKA knockdown decreased the number of acellular capillaries in diabetic retinas (Figure 4C).

To validate these findings, we further employed a pharmacological approach using MN58b to replicate the effects of CHKA inhibition. Mice received weekly intravitreal injections of 50 µm MN58b or DMSO as a vehicle control for 2 weeks. Evans blue assays and PAS staining were consistent with those obtained from the AAV‐mediated CHKA knockdown. Specifically, treatment with MN58b significantly reduced retinal vascular leakage and the formation of acellular capillaries compared to DMSO‐treated and untreated DR mice (Figure 4D,E). Collectively, these results demonstrate that targeting CHKA activity, either through genetic knockdown or pharmacological inhibition, alleviates retinal vascular dysfunction.

### 2.5 Transcriptomic and Metabolomic Analyses reveal Altered Gene Expression and NAD^+^ Metabolism Following CHKA Silencing

**Figure 5:**
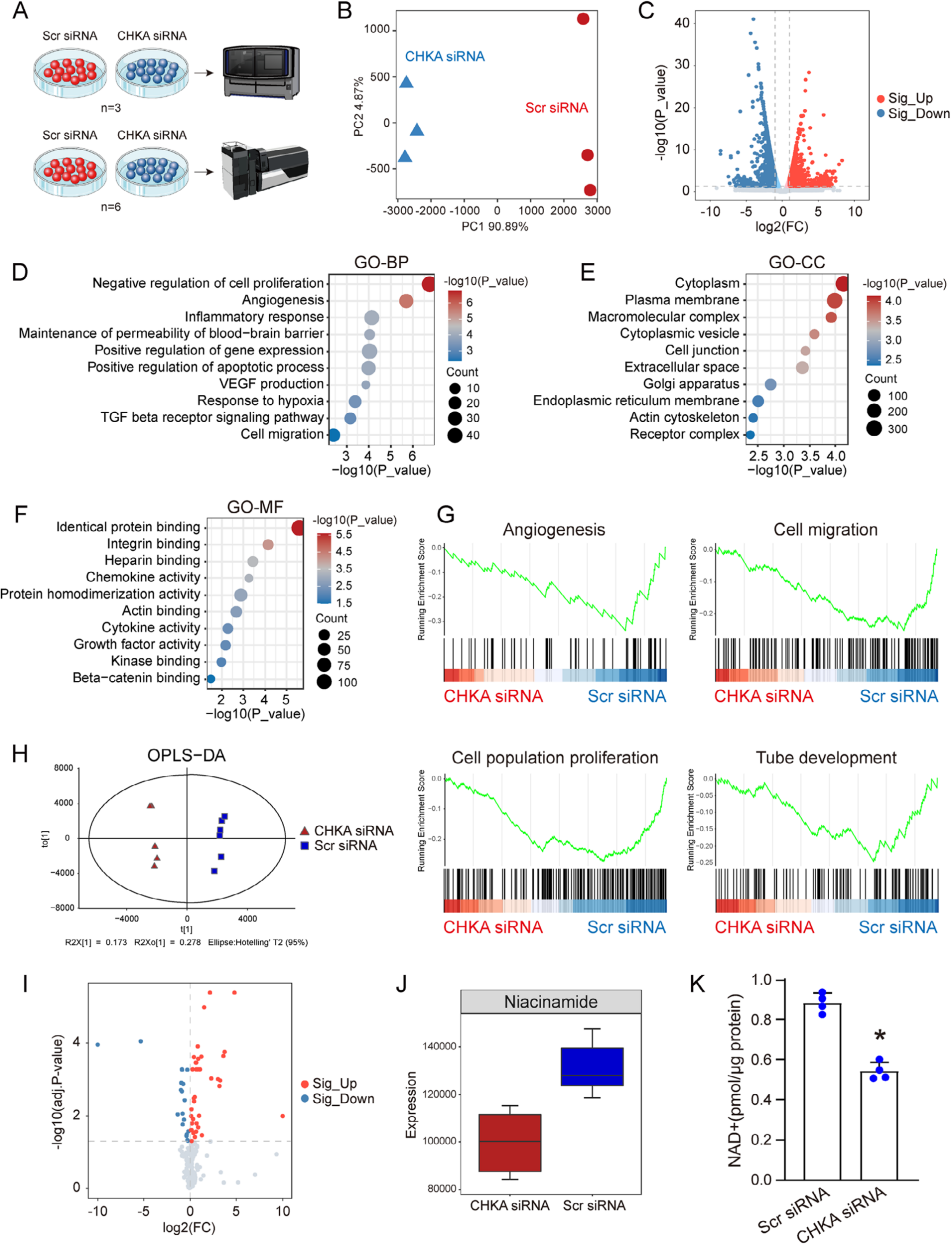
Transcriptomic and metabolomic analyses reveal gene expression and NAD^+^ metabolism changes following CHKA silencing. A) Schematic illustration of the experimental workflow for transcriptomic and metabolomic analyses of HRMECs transfected with CHKA siRNA or Scr siRNA. B) PCA of RNA‐seq data from HRMECs transfected with CHKA siRNA and Scr siRNA (n = 3), with each replicate represented by a triangle or dot. C) Volcano plot displaying the DEGs between CHKA siRNA and Scr siRNA group. D‐F) Dot plots showing the GO‐BP, GO‐CC, and GO‐MF enrichment analyses for the top 1000 DEGs between the CHKA siRNA and Scr siRNA groups. G) Representative GSEA plots illustrating the downregulation of specific terms in the CHKA siRNA group compared to Scr siRNA group. H) Metabolite profiles of HRMECs transfected with CHKA siRNA and Scr siRNA (n = 6) analyzed by OPLS‐DA, with each replicate depicted as a triangle or square. I) Volcano plot illustrating the differential metabolites between CHKA siRNA and Scr siRNA group. J) Box plot showing the expression levels of niacinamide in HRMECs treated with CHKA siRNA and Scr siRNA. K) Bar graph representing NAD^+^ levels in HRMECs transfected with CHKA siRNA and Scr siRNA. n = 4; *P < 0.05; Student's *t* test.

To elucidate the molecular changes induced by CHKA silencing, RNA sequencing (RNA‐seq) and GC‐MS‐based untargeted metabolomic analyses were performed to evaluate changes in gene expression and metabolic profiles following CHKA silencing (**Figure** **5**A). Principal component analysis (PCA) was performed to examine the relationships between all six RNA‐seq samples. The results indicated that samples within each group clustered closely with similar characteristics, while the CHKA siRNA and Scr siRNA groups were distinctly separate (Figure 5B), confirming the reliability and reproducibility of RNA‐seq dataset. Using cutoff thresholds of |log2(FC)| > 1 and *P* value < 0.05, differential expression analysis identified 2190 differentially expressed genes (DEGs) between CHKA siRNA‐transfected and Scr siRNA‐transfected HRMECs, including 1109 up‐regulated genes and 1081 down‐regulated genes (Figure 5C). GO enrichment analysis of the DEGs revealed significant enrichment in terms related to cell proliferation, angiogenesis, cell migration, and cell adhesion following CHKA silencing (Figure 5D–F). Gene Set Enrichment Analysis (GSEA) further supported these findings, demonstrating that pathways such as “Angiogenesis (*P*.adjust: 0.01, NES: ‐1.98),” “Cell migration (*P*.adjust: 0.02, NES: ‐1.86),” “Cell population proliferation (*P*.adjust: 0.001, NES: ‐2.09),” and “Tube development (*P*.adjust: 0.02, NES: ‐1.68)” were significantly down‐regulated in CHKA siRNA group compared to Scr siRNA group (Figure 5G).

Metabolomic profiling using the Orthogonal Partial Least Squares Discriminant Analysis (OPLS‐DA) demonstrated a clear distinction between CHKA siRNA group and Scr siRNA group (Figure 5H). By applying the cutoff thresholds set of VIP > 1.0 and adjusted *P* value < 0.05, 59 altered metabolites were identified, including 40 up‐regulated and 19 down‐regulated metabolites (Figure 5I). Among the down‐regulated metabolites, the top 10 with the highest VIP scores were primarily associated with choline metabolism, such as glycerophosphocholine, phosphatidylethanolamine‐N‐methylated, and phosphatidylcholine, which are closely linked to CHKA function. We also observed a significant reduction in niacinamide (NAM) levels in CHKA siRNA group (Figure 5J). NAM is a critical precursor in NAD^+^ salvage pathway and serves as a direct product of NAD^+^ consumption and degradation. The reduction in NAM levels suggests impaired NAD^+^ metabolism, which could contribute to the observed alteration in endothelial function. NAD^+^ levels were detected by the NAD^+^ assay kit, revealing a significant decrease in NAD^+^ levels in CHKA siRNA‐transfected HRMECs compared to Scr siRNA‐transfected cells (Figure 5K). Collectively, transcriptomic and metabolomic analyses demonstrate that CHKA silencing significantly disrupts gene expression related to angiogenesis and alters NAD^+^ metabolism, highlighting the role of CHKA in endothelial angiogenic effects.

### 2.6 Restoration of NAD^+^ Levels Partially Rescues Endothelial Angiogenic Effects In Vitro and In Vivo

**Figure 6:**
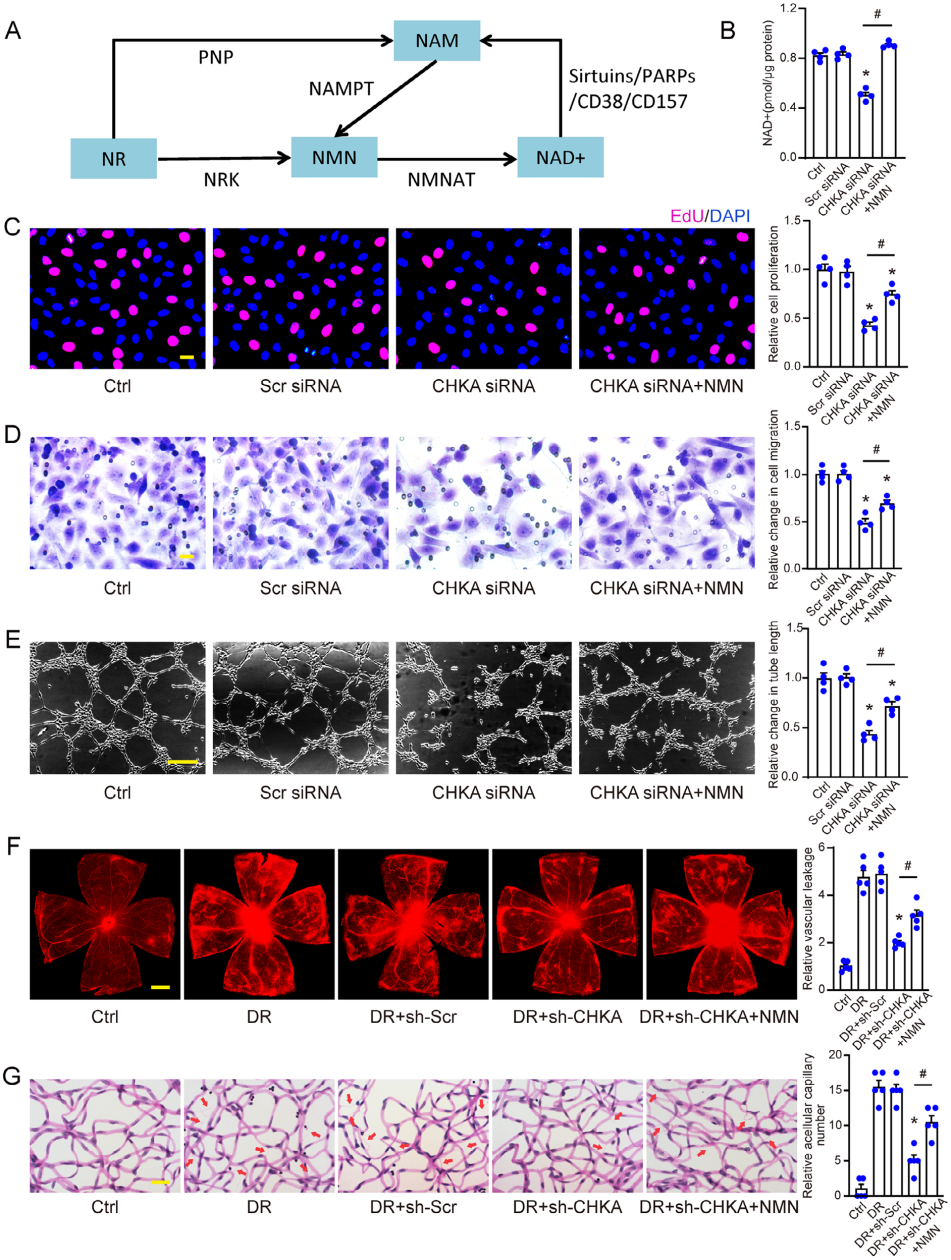
Restoration of NAD^+^ levels partially rescues endothelial angiogenic effects in vitro and in vivo. A) Schematic diagram depicting the mammalian NAD^+^ salvage pathway, with main metabolites represented in boxes. NR: nicotinamide riboside; NMN: nicotinamide mononucleotide; NRK: nicotinamide riboside kinase (1 and 2); PNP: purine nucleoside phosphorylase; NAMPT: nicotinamide phosphoribosyltransferase; NMNAT: nicotinamide mononucleotide adenylyltransferase (1, 2, and 3); PARPs: poly (ADP‐ribose) polymerases. HRMECs were transfected with Scr siRNA, CHKA siRNA, or left untreated as controls. At 24 h post‐transfection with CHKA siRNA, the medium was replaced by fresh medium supplemented with 0.5 mm NMN. B) NAD^+^ levels were determined for each group using an NAD^+^ assay kit. C) Cell proliferation was assessed by EdU staining. Scale bar: 20 µm. D) Migration capability was measured by the Transwell migration assay. Scale bar: 20 µm. E) Tube formation was analyzed with a tube formation assay. Scale bar: 100 µm. n = 4; *P < 0.05 compared to Scr siRNA group; ^#^ *P* < 0.05; One‐way ANOVA with Bonferroni post hoc test. DR mice received intravitreal injections of sh‐Scr AAV, sh‐CHKA AAV, or left untreated (DR), with age‐matched non‐diabetic mice taken as Ctrl. Following 4‐week treatment, sh‐CHKA AAV‐injected mice were subjected to daily intraperitoneal injections of NMN (500 mg kg^−1^ body weight), or equivalent volume of PBS for 2 weeks. F) Retinal vascular leakage was evaluated by Evans blue assay. Scale bar: 500 µm. Trypsin digestion followed by PAS staining (G) identified acellular capillaries in the retina (indicated using red arrows). Scale bar: 10 µm. n = 5; *P < 0.05 compared to DR+sh‐Scr group; ^#^ *P* < 0.05; One‐way ANOVA with Bonferroni post hoc test.

To determine whether CHKA regulates endothelial angiogenic effects through NAD⁺ metabolism, we conducted experiments to assess whether restoring NAD^+^ levels could reverse the effects of CHKA silencing on endothelial angiogenic effects. **Figure** **6**A presents a diagram of mammalian NAD^+^ salvage pathway, highlighting key metabolites involved in maintaining cellular NAD^+^ levels. As nicotinamide mononucleotide (NMN) is a direct precursor of NAD^+^, its supplementation offers a rational method to restore NAD^+^ levels. Previous studies have shown that NMN supplementation is an effective method for replenishing NAD^+^. In this study, HRMECs were transfected with CHKA siRNA, and 24 h post‐transfection, the culture medium was replaced with fresh medium containing 0.5 mm NMN to evaluate its effect on NAD^+^ restoration. NMN supplementation increased NAD^+^ levels in CHKA siRNA‐transfected cells, restoring NAD^+^ to levels comparable to those of the control group (Figure 6B). Subsequently, we investigated whether restoring NAD^+^ levels through NMN supplementation could rescue the suppressed angiogenic effects induced by CHKA silencing in HRMECs. NMN supplementation partially rescued endothelial cell proliferation, migration, and tube formation, which had been reduced due to CHKA silencing. EdU staining demonstrated that NMN treatment increased cell proliferation (Figure 6C), while transwell migration (Figure 6D) and tube formation assays (Figure 6E) revealed enhanced migration and capillary‐like structure formation, respectively. These results indicate that replenishing NAD^+^ via NMN supplementation can reduce the inhibitory effects of CHKA silencing on endothelial angiogenic effects in vitro.

To confirm these findings in vivo, we used a diabetic murine model to evaluate the effects of NMN supplementation on retinal vascular dysfunction. Diabetic mice injected intravitreally with CHKA shRNA AAV were administered daily intraperitoneal injections of NMN or PBS. NMN supplementation increased retinal vascular leakage, as measured by Evans blue assays (Figure 6F), and promoted acellular capillary formation, as shown by trypsin digestion and PAS staining (Figure 6G). These findings suggest that NMN supplementation partially restores the suppressed pro‐angiogenic effects following CHKA silencing. Collectively, these results support the hypothesis that CHKA regulates endothelial angiogenic effects through NAD^+^ metabolism in vitro and in vivo.

### 2.7 CHKA Regulates Endothelial Cell Function and Angiogenesis through NAD^+^‐SIRT1‐Notch Signaling Axis

**Figure 7:**
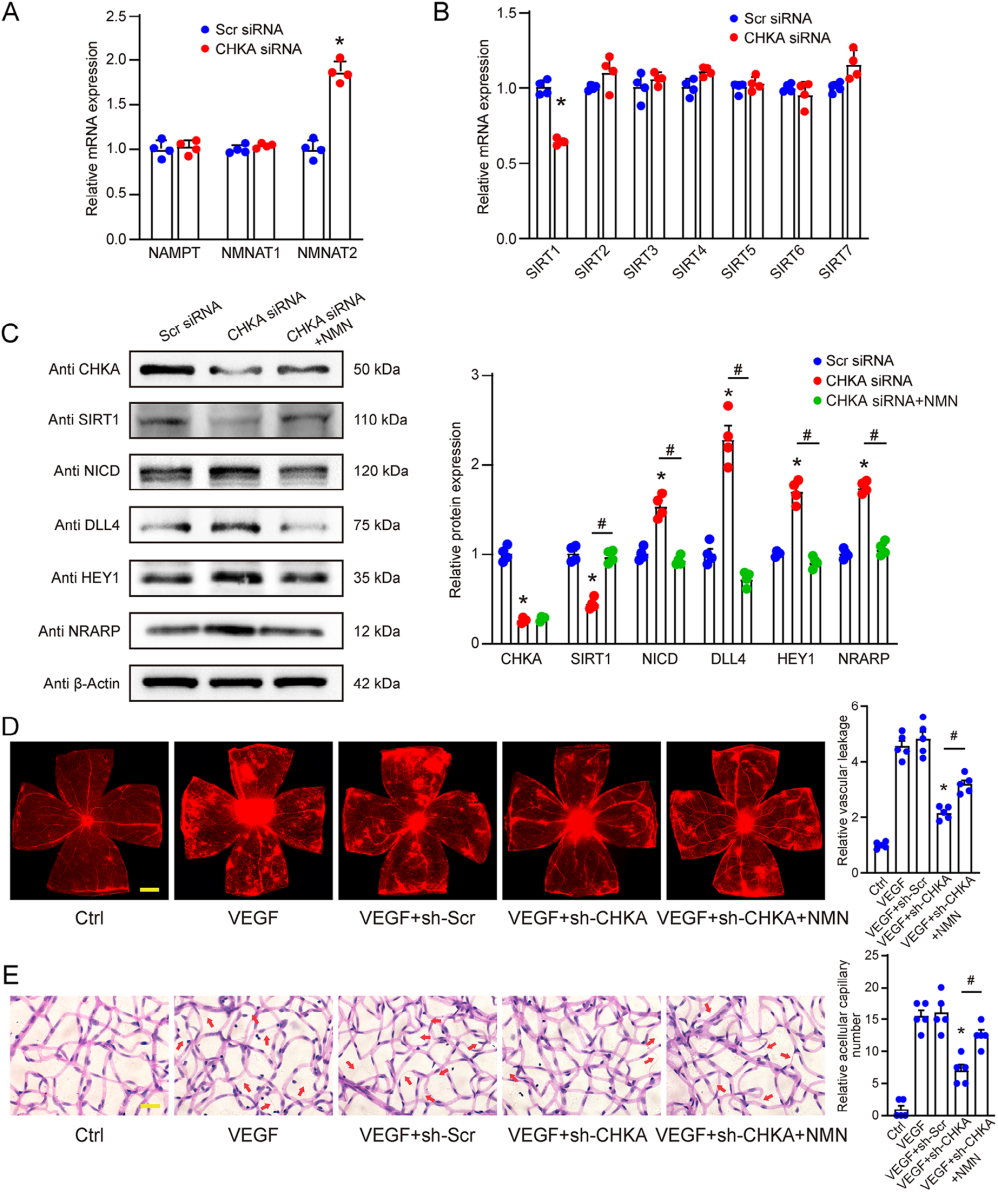
CHKA regulates endothelial cell function and angiogenesis through NAD^+^‐SIRT1‐Notch signaling axis. A) Relative mRNA expression levels of key enzymes in NAD^+^ salvage pathway, including NAMPT, NMNAT1, and NMNAT2, in HRMECs transfected with Scr siRNA or CHKA siRNA. Cells were harvested 24 h post‐transfection for qPCR assay. B) Relative mRNA expression levels of Sirtuins family members (SIRT1‐7) in HRMECs transfected with Scr siRNA or CHKA siRNA. Cells were harvested 24 h post‐transfection for qPCR assay. n = 4; *P < 0.05 compared to Scr siRNA; Student's *t* test. C) Western blot and densitometric analyses showing protein expression levels of CHKA, SIRT1, and Notch pathway‐related proteins (NICD, DLL4, HEY1, and NRARP) in HRMECs transfected with Scr siRNA, CHKA siRNA, or CHKA siRNA followed by NMN supplementation. β‐actin was detected as the loading control. n = 4; *P < 0.05; ^#^ *P* < 0.05; One‐way ANOVA with Bonferroni post hoc test. D, E) Mice received intravitreal injections of sh‐Scr AAV, sh‐CHKA AAV, or were left untreated. After 4 weeks, VEGF was injected intravitreally, and NMN or PBS was administered daily via intraperitoneal injection for 2 weeks. Age‐matched healthy mice were taken as Ctrl. Representative images of retinal flat mounts stained with Evans blue to assess retinal vascular leakage. Scale bar: 500 µm (D). Trypsin digestion followed by PAS staining identified acellular capillaries in the retina (indicated using red arrows). Scale bar: 10 µm (E). n = 5; *P < 0.05 compared to VEGF+sh‐Scr group; ^#^ *P* < 0.05; One‐way ANOVA with Bonferroni post hoc test.

To explore the role of CHKA in regulating endothelial function and angiogenesis via NAD⁺ metabolism, we analyzed the expression of key enzymes involved in NAD⁺ salvage pathway. qPCR assays revealed a significant up‐regulation of NMNAT2 expression following transfection of CHKA siRNA, whereas NAMPT and NMNAT1 levels remained unchanged compared to Scr siRNA group (**Figure** **7**A), suggesting a compensation for the reduced NAD^+^ levels caused by CHKA silencing via up‐regulating NMNAT2, a key enzyme in NAD^+^ synthesis. Given that NAD^+^ is a crucial cofactor for Sirtuins, which are a family of NAD^+^‐dependent deacetylases involved in regulating various cellular processes, we next analyzed the expression of the Sirtuins family members (SIRT1‐7) to determine the effects of NAD^+^ decrease on these enzymes. The results revealed a marked reduction in SIRT1 expression following CHKA silencing, while the levels of SIRT2‐7 remained unchanged (Figure 7B), suggesting a crucial role for SIRT1 in mediating the effects of CHKA on endothelial function. To further investigate the downstream effects of SIRT1 and its potential role in angiogenic effects, we focused on Notch signaling pathway, a key regulator of cell fate.^[^ ^28^, ^29^ ^]^ It is well‐established that Notch signaling activation can inhibit angiogenesis. SIRT1 can directly regulate the stability and activity of Notch1 intracellular domain (NICD) through deacetylation, thereby regulating Notch signaling.^[^ ^30^ ^]^ Thus, we analyzed whether Notch pathway was involved in mediating the effects of CHKA silencing on endothelial function. We performed western blot analysis to detect the protein levels of CHKA, SIRT1, and Notch pathway‐related proteins, including NICD, DLL4, HEY1, and NRARP. CHKA silencing effectively decreased CHKA and SIRT1 protein levels. The reduction in SIRT1 levels was accompanied by increased levels of NICD, DLL4, HEY1, and NRARP, suggesting that Notch signaling was activated upon CHKA silencing. However, NMN supplementation restored SIRT1 levels and reduced the levels of NICD, DLL4, HEY1, and NRARP expression, suggesting that NAD⁺ replenishment can inhibit Notch pathway activation induced by CHKA silencing (Figure 7C).

VEGF is a key factor of pathological angiogenesis, and Notch pathway can regulate endothelial sensitivity to VEGF signaling. To investigate whether CHKA affects endothelial dysfunction through the NAD⁺‐SIRT1‐Notch axis, we employed a VEGF‐induced retinal vascular leakage model. EB assays revealed that VEGF injection significantly increased vascular leakage compared to the control group. CHKA silencing reduced VEGF‐induced leakage, whereas NMN supplementation in CHKA silencing mice partially restored the leakage (Figure 7D). PAS staining of retinal trypsin‐digested preparations further showed that CHKA silencing reduced the number of acellular capillaries induced by VEGF, while NMN supplementation partially reversed this effect, increasing the number of acellular capillaries (Figure 7E). These findings suggest that CHKA regulates endothelial function and angiogenesis through the NAD⁺‐SIRT1‐Notch signaling axis.

### 2.8 Clinical Significance of CHKA in Diabetic Retinopathy

**Figure 8:**
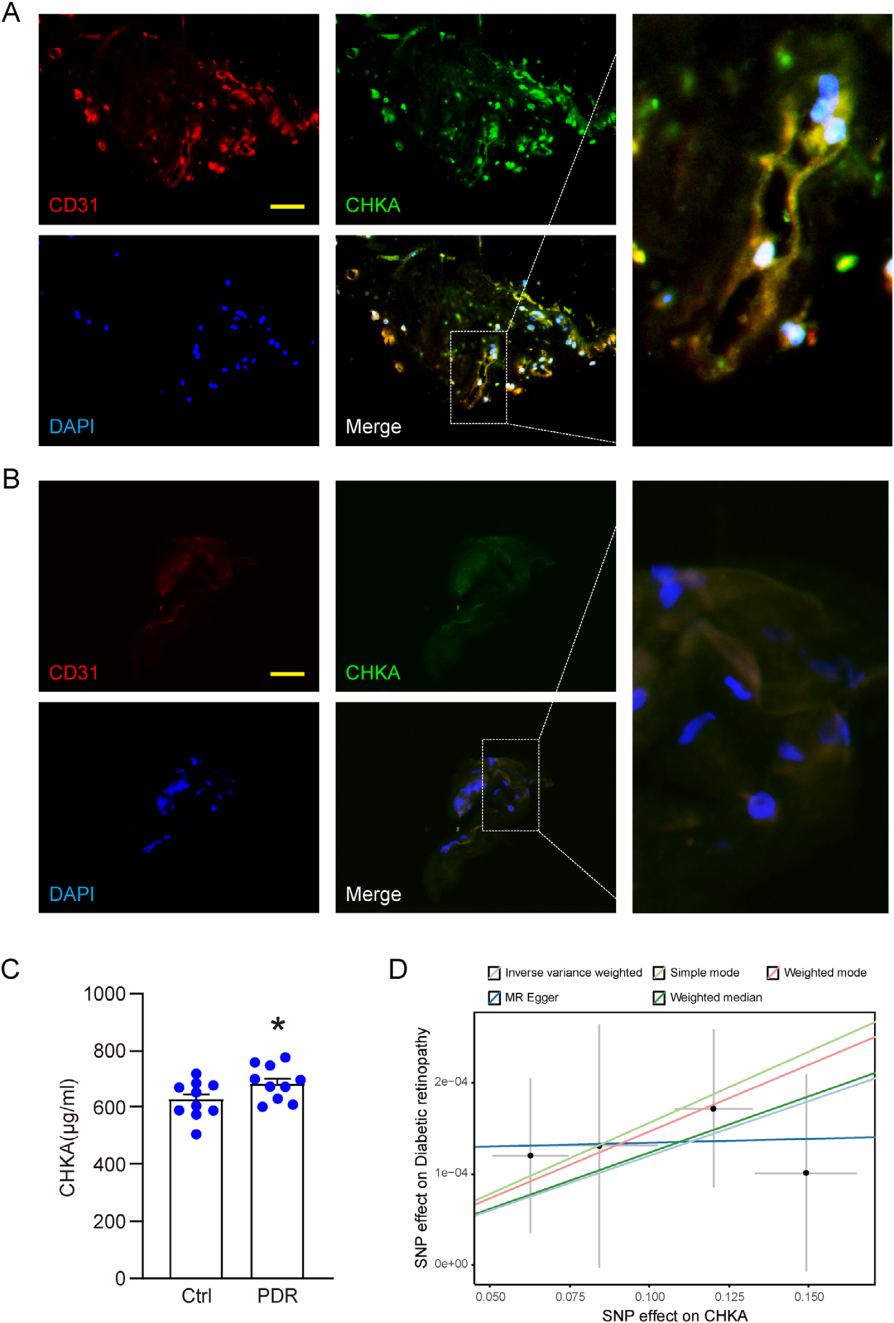
Clinical significance of CHKA in diabetic retinopathy. A) Immunofluorescence staining of proliferative membranes from proliferative diabetic retinopathy (PDR) patients. Samples were double‐stained for CD31 (red) and CHKA (green), with nuclei counterstained using DAPI (blue). Scale bar: 50 µm. B) Immunofluorescence staining of clinical samples from patients with epiretinal membranes (ERMs). The epiretinal membranes were stained for CD31 (red) and CHKA (green). Scale bar: 50 µm. C) ELISA measurement of CHKA levels in aqueous humor of PDR patients (n = 10) compared to age‐matched cataract patients (n = 10) as controls. *P < 0.05; Student's *t* test. D) Mendelian randomization (MR) analysis evaluating the effect of CHKA expression on DR risk, using single nucleotide polymorphisms (SNPs) as instrumental variables. The SNP effect on CHKA (exposure) and DR (outcome) was evaluated by five MR methods: inverse variance weighted, MR Egger, simple mode, weighted mode, and weighted median.

To further investigate clinical significance of CHKA in DR, we examined its expression in human proliferative diabetic retinopathy (PDR) samples. Immunofluorescence staining of fibrovascular membrane from PDR patients revealed co‐localization of CHKA with endothelial marker CD31, showing the elevated expression of CHKA in vascular regions undergoing pathological angiogenesis (**Figure** **8**A). In contrast, staining of epiretinal membranes (ERMs) showed minimal expression of both CHKA and CD31 (Figure 8B). These findings suggest that CHKA up‐regulation is largely confined to pathological vascular tissues in PDR.

We next investigated whether CHKA levels in aqueous humor reflect disease status. ELISA analysis showed significantly higher CHKA concentrations in aqueous humor from PDR patients compared to the age‐matched cataract controls (Figure 8C), suggesting a potential link between elevated CHKA levels and DR progression.

To explore the genetic contribution of CHKA to DR, we performed a Mendelian Randomization (MR) analysis using single nucleotide polymorphisms (SNPs) associated with CHKA expression as instrumental variables. This approach minimizes confounding and reverse causality common in observational studies. Five MR methods—including inverse variance weighted (IVW), MR Egger, simple mode, weighted mode, and weighted median—consistently demonstrated a positive association between genetically predicted CHKA expression and increased DR risk (Figure 8D; Figure S6A, Supporting Information). Notably, IVW method indicated a significant causal relationship, supporting CHKA's contribution to DR pathogenesis.

Sensitivity analyses, including heterogeneity testing (Figure S6B, Supporting Information), pleiotropy assessment (Figure S6C, Supporting Information), and leave‐one‐out analysis (Figure S6D, Supporting Information), further validated the robustness of our findings. The forest plot (Figure S6E, Supporting Information) shows consistent effect estimates across individual SNPs. These results underscore the clinical significance of CHKA in DR and highlight its potential as a therapeutic target and biomarker.

## 3 Discussion

Cellular heterogeneity, the existence of distinct cell subpopulations within the same tissue, is a fundamental biological phenomenon.^[^ ^31^, ^32^ ^]^ These variations can stem from genetic differences or responses to external stimuli, which is critical for both normal physiology and disease pathogenesis.^[^ ^33^, ^34^ ^]^ ScRNA‐seq is a powerful method for studying cellular heterogeneity at unprecedented resolution. Unlike traditional transcriptomic methods, which masks cell‐to‐cell variation, scRNA‐seq enables the analysis of gene expression at the individual cell level, revealing distinct cellular identities and rare subpopulations. This technology has revolutionized fields such as oncology and neuroscience. In cancer research, scRNA‐seq has unraveled the complexity of tumor microenvironments and identified therapy‐resistant cell populations. In neuroscience, it has facilitated the classification of diverse neural cell types and elucidated their contributions to neurodegenerative disorders.^[^ ^35^, ^36^ ^]^ However, its application in ophthalmology remains nascent. Further studies are required to address complex ocular conditions, such as retinal diseases.

DR is one of the most prevalent complications of diabetes. As a major cause of vision loss worldwide, DR imposes a substantial burden on both individuals and healthcare systems. The pathological mechanisms underlying DR are highly complex, involving multiple factors such as inflammation, oxidative stress, and vascular dysfunction.^[^ ^37^ ^]^ Among these pathological features, the hallmark of the most prominent feature of DR is pathological retinal neovascularization, which can lead to severe complications such as vitreous hemorrhage and tractional retinal detachment.^[^ ^38^, ^39^ ^]^ Consequently, anti‐angiogenic therapies targeting ECs have emerged as a therapeutic strategy for DR, primarily through inhibition of pathological neovascularization.

EC dysfunction represents both an initiating event and a key driver in DR progression, critically contributing to pathological neovascularization.^[^ ^40^ ^]^ Dysfunctional ECs not only contribute to increased vascular permeability but also promote aberrant neovascularization. Given the critical role of ECs in DR pathogenesis, they have emerged as one of the primary therapeutic targets. Thus, studying the transcriptional and functional heterogeneity of ECs under diabetic conditions using scRNA‐seq is essential. To elucidate the mechanism underlying EC dysfunction, we used scRNA‐seq to profile transcriptional heterogeneity in retinal ECs from both healthy and DR mice. Three distinct EC subpopulations were identified, which exhibited marked transcriptional differences. Functional enrichment analysis identified one subcluster, characterized by elevated CHKA expression, appeared to be closely associated with angiogenic functions. Comparative analysis demonstrated significant up‐regulation of CHKA in this subcluster within DR samples compared to controls. These results implicate CHKA in pathological neovascularization, suggesting its potential as a biomarker for angiogenic EC subpopulations and a novel therapeutic target for anti‐angiogenic strategies.

CHKA is an enzyme that plays a critical role in phospholipid biosynthesis. It catalyzes the conversion of free choline to phosphocholine, the rate‐limiting step in phosphatidylcholine (PC) synthesis. CHKA can also phosphorylate ethanolamine, participating in phosphatidylethanolamine (PE) production. Thus, CHKA is essential not only for membrane phospholipid biogenesis but also for regulating energy metabolism under cellular stress conditions. In diabetes, the relationship between ECs and energy metabolism undergoes significant alterations, which form the pathological basis for multiple diabetic complications, particularly DR, diabetic nephropathy, and cardiovascular diseases. As the major phospholipid component of endothelial membranes, PC is crucial for maintaining membrane fluidity, integrity, and selective permeability. CHKA dysregulation disrupts PC homeostasis, leading to membrane destabilization and altered lipid composition. Compromised endothelial barrier function manifests as vascular leakage and edema—hallmarks of microvascular complications like DR and diabetic nephropathy. Furthermore, PC metabolism disturbances promote oxidized phospholipid (OxPC) accumulation, triggering inflammatory cascades and macrophage adhesion that exacerbate macrovascular pathologies such as atherosclerosis. These multifaceted functions of CHKA thus position it as a potential key regulator in diabetic vascular complications.

CHKA has been extensively studied in the fields of cancers, where it is implicated in promoting tumor cell proliferation, migration, and survival. Elevated CHKA expression has been linked to poor prognosis in various malignancies, suggesting its role as a potential oncogene.^[^ ^41^, ^42^ ^]^ In this study, we investigated the effect of CHKA silencing at both cellular and animal levels. We demonstrated that CHKA silencing in ECs significantly impaired their proliferation, migration, and tube formation ability. In DR mice, CHKA knockdown reduced pathological retinal vascular dysfunction. These results suggest that CHKA is involved in regulating endothelial function and angiogenic process.

To further elucidate the underlying mechanism, we conducted metabolomic and transcriptomic analyses on CHKA‐silenced ECs. CHKA knockdown could disrupt NAD^+^ metabolism and decreased the activity of NAD^+^‐dependent deacetylase SIRT1. SIRT1 is a critical regulator of several biological processes, including angiogenesis, and exerts its effects through the deacetylation of various protein substrates.^[^ ^43^ ^]^ One key target of SIRT1 in ECs is the NICD, the active form of Notch receptor that translocates to the nucleus to regulate gene expression. In particular, SIRT1 deacetylates NICD at specific lysine residues, which affects both its stability and transcriptional activity, thereby regulating Notch signaling pathway.^[^ ^30^, ^44^ ^]^ Western blot analysis demonstrated that CHKA silencing significantly reduced SIRT1 protein levels, leading to diminished deacetylation of NICD and consequent activation of Notch signaling pathway. Importantly, supplementation with NMN partially rescued the anti‐angiogenic effects induced by CHKA knockdown. This intervention restored SIRT1 expression and attenuated Notch pathway activation. These results establish that CHKA regulates EC function and pathological angiogenesis through the NAD^+^‐SIRT1‐Notch signaling axis. The Notch pathway is well known for its anti‐angiogenic effects through the regulation of downstream target genes, but it also regulates VEGF signaling pathway. Activation of Notch signaling exerts a feedback inhibition on VEGF receptor expression, reducing the sensitivity of ECs to VEGF stimulation.^[^ ^45^ ^]^ To investigate CHKA's role in VEGF‐mediated vascular pathology, we used VEGF‐induced retinal vascular leakage model. CHKA knockdown reduced VEGF‐induced vascular leakage and acellular capillary formation, while NMN supplementation partially reversed these effects. These findings confirm CHKA's critical involvement in VEGF‐dependent vascular dysfunction, and further support the therapeutic potential of targeting NAD^+^‐SIRT1‐Notch axis.

We also investigated the clinical relevance of CHKA dysregulation in DR. We demonstrated elevated CHKA expression in the vascular regions of PDR membranes, while minimal CHKA expression was observed in ERMs. To further validate the clinical significance of CHKA in DR, we analyzed aqueous humor samples from cataract (control) and PDR patients using ELISA. These results confirmed elevated CHKA levels in PDR patients. To establish a potential causal relationship, we also conducted MR analysis, which revealed that genetically predicted higher CHKA expression was positively associated with increased DR risk. This genetic evidence suggests that CHKA plays a causative role in DR development.

In conclusion, this study offers important insights into the heterogeneity of retinal ECs and identifies CHKA as a critical regulator of retinal vascular dysfunction in DR. This underscores the potential of CHKA both as a promising therapeutic target and as a predictive biomarker for DR.

## 4 Experimental Section

### Single‐Cell Library Construction and Sequencing

After euthanasia, mouse eyes were enucleated, and retinas were isolated for single‐cell suspension preparation. Retinas were dissected in cold PBS buffer and minced into small fragments on ice. For enzymatic digestion, retinal fragments were incubated in a papain digestion solution, which consisted of 700 µL reagent‐grade water, 100 µL freshly prepared 50 mm L‐Cysteine (Sigma‐Aldrich, USA), 100 µL 10 mm EDTA, 10 µL 60 mm 2‐mercaptoethanol (Sigma‐Aldrich, USA), and 1 mg/mL papain (Worthington, USA). The tissue was digested at 37 °C for 30 min. After digestion, the suspension was passed through a 70‐µm cell strainer (Corning, USA) to remove tissue debris, followed by red blood cell depletion.

The single‐cell suspensions were analyzed on the BD Rhapsody scRNA‐seq platform (BD Biosciences, USA). After labeling and washing, cells were isolated in microwells, lysed, and the polyadenylated mRNAs were captured using barcoded beads. Unique molecular identifiers (UMIs) were incorporated during reverse transcription. Whole transcriptome amplification and library construction were performed according to the BD Rhapsody workflow. Libraries were sequenced using the Illumina 10X platform (Illumina, USA). Raw reads underwent quality control, annotation, cell identification, and were compiled into an expression matrix. RSEC and DBEC algorithms further refined the matrix.

### Single‐Cell Sequencing Data Analysis

Single‐cell RNA sequencing data was analyzed using the Seurat R package (version 4.3.0) to ensure quality control, cell clustering, and batch effect correction. UMIs were employed to remove PCR duplicates. The nCount threshold was set between 500 and 100 000 UMI counts per cell. Cells expressing <200 or >8000 genes, or exhibiting >10% mitochondrial UMI reads were excluded. Following quality control, 40574 cells remained for analysis. To reduce dimensionality, PCA was performed on the top 2000 hypervariable genes. Batch effects were corrected using the “harmony” package in R. Cell clustering was achieved using Seurat's shared nearest neighbor (SNN) modularity optimization algorithm. For cell type annotation, DEGs were identified in each cluster using “FindAllMarkers,” then manually assigned identities by assessing marker gene expression patterns, referencing literature, and the CellMarker database (http://117.50.127.228/CellMarker). Endothelial subclusters were identified via “FindClusters.” Cellular distributions were visualized using UMAP.

For functional analyses, gene list was subjected to GO enrichment analysis, including biological processes (BP), cellular components (CC), and molecular functions (MF). Pathway enrichment analyses were performed using KEGG and REACTOME databases. These analyses were performed using the DAVID Bioinformatics Database (DAVID Bioinformatics Resources, https://david.ncifcrf.gov/).

### Animals

All animal experiments followed institutional IACUC guidelines (Approval No. IACUC‐2410049) and complied with the ARVO Statement for Animal Use in Ophthalmic Research. C57BL/6J mice were obtained from Nanjing Junke Bioengineering Co., Ltd. (Nanjing, China). Mice were maintained under controlled conditions (12 h light‐dark cycle, 40–60% humidity, 20–25 °C) with ad libitum food/water access. Health status was routinely monitored, and all procedures employed appropriate anesthesia to minimize discomfort.

### STZ‐Induced Diabetic Model

Male C57BL/6J mice (6‐8 weeks, 20–25 g) were fasted for 12 h before receiving intraperitoneal injections of STZ (50 mg kg^−1^, dissolved in 0.1 m citrate buffer, pH 4.5) for five consecutive days. Control mice were injected with citrate buffer alone. Diabetes was confirmed 7 days after the final injection by measuring random blood glucose levels, with levels above 16.7 mm considered diabetic. Diabetic mice were maintained under standard conditions with free access to food and water, and fasting blood glucose levels were monitored monthly. At the 6‐month endpoint, representative diabetic mice (n = 6) exhibited an average body weight of 19.2 ± 1.6 g and HbA1c level of 9.5 ± 0.8%.

### Oxygen‐Induced Retinopathy (OIR) Model

The OIR model was established using C57BL/6J mouse pups. On postnatal day 7 (P7), the pups along with their nursing mothers were placed in a controlled oxygen chamber with 75% oxygen for 5 days to induce hyperoxia. On postnatal day 12 (P12), the mice were returned to room air for 5 days, inducing retinal hypoxia‐driven neovascularization. Retinal tissues of mouse pups were collected on postnatal day 17 (P17) for further analysis.

### Evans Blue (EB) Assay

EB assays were performed to assess retinal vascular leakage. EB dye (30 mg mL^−1^, KEHBIO Technology, China) was prepared by dissolving in saline and left overnight on a shaker table. The solution was then filtered through a 0.45‐µm filter to ensure its clarity. Mice were anesthetized before the femoral vein was exposed through a small incision on the inner thigh. Using an insulin syringe, EB dye was injected into the femoral vein at a dose of 45 mg kg^−1^. After injection, the mice were placed on a rewarming table for ≈30 min to allow the dye to circulate throughout the body. Following circulation, the mice were euthanized, and their eyeballs were enucleated and fixed in 4% PFA for 50 min. Retinas were dissected, flat‐mounted, and assessed for vascular leakage via fluorescence microscopy (Olympus, Japan). The quantification of EB dye was performed following established protocols.^[^ ^46^ ^]^

### Cell Culture and Transfection

HRMECs were cultured in Endothelial Cell Medium (ECM, Sciencell, USA), supplemented with 10% fetal bovine serum (FBS), 20 µg mL^−1^ endothelial cell growth supplement (ECGS), and 1% penicillin/streptomycin (P/S) in a humidified incubator set at 37 °C with 5% CO₂. CHKA‐KO HRMECs were maintained under the same standardized culture conditions as the wild‐type cells. For gene knockdown experiments, siRNAs targeting CHKA were obtained from Ribobio, China. Transfections were performed following the manufacturer's protocol for Lipofectamine RNAiMAX (Invitrogen, USA). Scr siRNA (Ribobio, China) was used as a negative control. Cells were incubated with siRNA complexes for 6 h, and the medium was replaced with fresh ECM. The sequence of CHKA siRNA was 5′‐GCAAGGTTTGATGCCTATT‐3′. For CHKA overexpression, HRMECs were transfected with pcDNA3.1 (empty vector) or pcDNA3.1‐CHKA (Ribobio, China) using Lipofectamine 3000 (Invitrogen, USA), with transfection efficiency verified by qPCRs.

### Clinical Sample Collection

This study adhered to the ethical principles outlined in the Declaration of Helsinki and the ARVO Statement on Human Subjects. Ethical approval was obtained from the Ethics Committee of the Affiliated Eye Hospital, Nanjing Medical University (Ethics Approval Number: [2023] Research Ethics Review No. (2023014)). Informed consent was obtained from all participants. Patients with a history of ocular trauma, prior vitrectomy, glaucoma, or other retinal diseases, as well as systemic conditions that could affect retinal health (excluding diabetes), were excluded from the study. Proliferative membrane samples and ERMs were collected from patients with PDR and ERM patients, respectively. Samples were fixed, sectioned, and processed for immunofluorescence staining according to previously described protocols. Aqueous humor samples were collected from PDR patients and age‐matched cataract patients and were stored at −80 °C until analysis. CHKA expression levels in the samples were measured using an ELISA kit (MEIMIAN, China).

### Statistical Analysis

Statistical analysis was performed using GraphPad Prism software (GraphPad Software, USA, version 9.4.1). Data is presented as mean ± SEM. For in vivo experiments, each n represents a single animal, while for in vitro experiments, each n corresponds to an independent experiment. Statistical significance between groups was determined using either a two‐tailed Student's *t* test or one‐way analysis of variance (ANOVA). *P* < 0.05 was considered statistically significant. All experiments were conducted at least four times to ensure robust and reliable results, with the number of replicates specified in each figure legend.

## Conflict of Interest

The authors declare no conflict of interest.

## Author Contributions

L.R., L.Z., Y.B., C.H., and X.L. contributed equally to this work. B.Y., Q.J., and X.C. designed research; L.R., L.Z., Y.B., C.H., X.L., F.M., and C.J. performed research; L.R., L.Z., Y.B., W.M., and M.Y. analyzed data; and L.R., L.Z., and B.Y. wrote the paper.

## Supporting information



Supporting Information

## Data Availability

The data that support the findings of this study are available from the corresponding author upon reasonable request.
