# Supplementary material for: Metabolic Stress‐Induced Choline Kinase α (CHKA) Activation in Endothelial Subpopulation Contributes to Diabetes‐Associated Microvascular Dysfunction
Source: Adv Sci (Weinh). 2025 Jun 23;12(33):e17045. doi: 10.1002/advs.202417045 (PMC12412581; doi:10.1002/advs.202417045)
Supplement: Supplementary file 1 — Supporting Information [file ADVS-12-e17045-s001.pdf]

ADVANCED SCIENCE
Open Access

Supporting Information

for *Adv. Sci.*, DOI 10.1002/advs.202417045

Metabolic Stress-Induced Choline Kinase α (CHKA) Activation in Endothelial Subpopulation Contributes to Diabetes-Associated Microvascular Dysfunction

Ling Ren, Linyu Zhang, Yun Bai, Chang Huang, Xiaosa Li, Fanfei Ma, Wan Mu, Mudi Yao, Chang Jiang, Xiangjun Chen, Qin Jiang* and Biao Yan**

ADVANCED SCIENCE
Open Access

Supporting Information

for *Adv. Sci.*, DOI 10.1002/advs.202417045

Metabolic Stress-Induced Choline Kinase α (CHKA) Activation in Endothelial Subpopulation Contributes to Diabetes-Associated Microvascular Dysfunction

Ling Ren, Linyu Zhang, Yun Bai, Chang Huang, Xiaosa Li, Fanfei Ma, Wan Mu, Mudi Yao, Chang Jiang, Xiangjun Chen, Qin Jiang* and Biao Yan**

Supporting Information

Supporting methods

Immunofluorescence Staining of Retinal Flat-mounts: Retinal flat-mounts were prepared by enucleating the eyes and fixing them in 4% paraformaldehyde (PFA) at room temperature. Retinas were carefully dissected and transferred to PBS, then cut into four quadrants. The retinas were then blocked and permeabilized in a blocking solution containing 1% Triton X-100 and 5% bovine serum albumin (BSA) in PBS for 1 h at 37°C. Primary antibodies were diluted in the blocking solution and incubated with the retinas overnight at 4°C. After washing in PBS with 0.1% Tween 20 (PBST), retinas were incubated with appropriate Alexa Fluor-conjugated secondary antibodies for 2 h at room temperature. Blood vessels were stained with isolectin B4 (IB4) for direct visualization. The retinas were then washed and mounted onto slides for imaging using a fluorescence microscope (Olympus, Japan).

Immunofluorescence Staining of Retinal Frozen Sections: After enucleation, the eyes were immediately fixed in 4% PFA at 4°C for 12 h. Following fixation, the samples were cryoprotected in a 30% sucrose solution for 36 h. The eyes were then embedded in optimal cutting temperature (OCT) compound and frozen at -80°C. Serial sections (10-12 μm) were cut using a cryostat and placed onto glass slides. The sections were then permeabilized for 30 min and blocked with 5% BSA for 1 h at room temperature. Primary antibodies were diluted in blocking solution and incubated with the sections overnight at 4°C. After washing with PBS, the sections were incubated

with Alexa Fluor-conjugated secondary antibodies for 2 h at room temperature in the dark. Nuclei were counterstained with DAPI. Fluorescence imaging was performed using a fluorescence microscope (Olympus, Japan).

Intravitreal Injection: After anesthesia and pupil dilation, a 30-gauge needle was used to create a limbal incision, avoiding lens and retinal damage. A 33-gauge Hamilton syringe (Hamilton Company, Reno, NV, USA) was then used to deliver intravitreal injections through this entry. The injection volumes were as follows: 1.5 μL for adeno-associated viruses (AAVs, PackGene Biotech, China), 2 μL for MN58b (50 μM, MCE, USA), and 2 μL fo VEGF (50 μM, ReproTech, USA). Care was taken to inject the solution slowly and prevent reflux by holding the needle in place for 30 sec post-injection. Following the injection, ofloxacin eye ointment was applied to prevent infection. The mice were then placed on a 37°C recovery platform until fully awake and mobile. The mice were regularly monitored until the designated time points for tissue collection and further experimental analysis.

Periodic Acid-Schiff (PAS) Staining: After euthanizing the mice, their eyes were enucleated and fixed for 24 h. The retinas were isolated and rinsed in double-distilled water overnight to remove any remaining fixative. Retinas were then subjected to trypsin digestion at 37°C for 1 h using a trypsin solution (BioFroxx, Germany) until flocculent tissues appeared. The excess tissue was gently flushed away using a 1 mL PBS-filled syringe, leaving only the retinal vascular network intact. The remaining vascular structures were then mounted on glass slides and air-dried. Once dry, the retinas were stained following the manufacturer's instructions using a PAS Stain Kit

(Solarbio, China). An inverted light microscope (Olympus, Japan) was used to capture pictures and count the number of acellular capillaries.

EdU Assay: Cell proliferation was detected by the BeyoClick EdU Cell Proliferation Kit (Beyotime, China). Briefly, HRMECs were seeded into 24-well plates and incubated with 10 μM EdU at 37°C for 2-3 h. After incubation, cells were fixed with 4% PFA for 15 min and permeabilized with 0.3% Triton X-100 for 10 min. Cells were then treated with the Azide Alexa Fluor 555 reaction solution for 30 min to visualize EdU incorporation and counterstained with Hoechst 33342. Images were captured by a fluorescent microscope (Olympus, Japan). The percentage of EdU-positive cells was calculated to determine cell proliferation.

Transwell Assay: HRMECs were suspended in 500 μL of serum-free medium and seeded into the upper chambers of Transwell inserts (8-μm pore size, Corning, USA). The lower chambers were filled with 500 μL of culture medium containing 10% FBS to serve as a chemoattractant. Cells were incubated at 37°C in a humidified incubator. After incubation, non-migrated cells on the upper surface of the insert were gently removed by a cotton swab. Migrated cells on the lower surface were fixed with 4% PFA for 10 min and then stained with 0.2% crystal violet for 30 min. The stained cells were imaged under an inverted light microscope (Olympus, Japan).

Wound Healing Assay: HRMECs grown in 6-well plates were wounded at optimal confluency using a 10-μL pipette tip. To eliminate proliferation effects, all wells were PBS-washed and serum-starved. After 24 h incubation, wound closure was quantified using an inverted microscope (Olympus, Japan).

Tube Formation Assay: Tube formation assays were performed to evaluate tube formation capacity of HRMECs. Briefly, a pre-cooled 24-well plate was coated with 50 μL of growth factor-reduced Matrigel (BD Biosciences, USA) each well and incubated at 37°C for 30 min to allow polymerization. HRMECs were then seeded onto the Matrigel and incubated at 37°C for 4-6 h. After incubation, images of tube-like structures were captured using an inverted light microscope (Olympus, Japan). Quantification of tube formation was performed using the Angiogenesis Analyzer plugin in ImageJ software.

Calcein-AM/PI Staining Assay: The apoptosis of HRMECs were detected using a Calcein-AM/PI Assay Kit (Beyotime, China). Briefly, HRMECs were seeded in a 24-well plate. After the required treatment, cells were washed with PBS and incubated with a staining solution containing Calcein-AM and propidium iodide (PI) at 37°C for 30 min in the dark. Following incubation, the cells were washed again with PBS, and images were captured using a fluorescence microscope (Olympus, Japan).

Quantitative Reverse Transcription PCR (qRT-PCR): Total RNAs were extracted from HRMECs using the FastPure Cell/Tissue Total RNA Isolation Kit (Vazyme, China). Reverse transcription of 1 μg RNA into cDNA was performed using the HiScript III RT SuperMix (Vazyme, China), with incubation at 37°C for 15 min followed by 85°C for 5 s. qRT-PCRs were performed using the ChamQ Universal SYBR qPCR Master Mix (Vazyme, China). PCR program was set to an initial denaturation at 95°C for 5 min, followed by 40 cycles of 10 s at 95°C and 30 s at 60°C. The relative expression levels of target genes were calculated using the $2^{-\Delta\Delta Ct}$ method.

Western Blot: Samples were lysed by the RIPA buffer containing protease inhibitors, followed by centrifugation at 13,000 × g for 30 min at 4°C. The supernatant was collected, and protein concentrations were quantified by the BCA Protein Assay Kit (Beyotime, China). Equal amounts of protein were subjected to SDS-PAGE and transferred onto PVDF membranes. Membranes were blocked at room temperature for 1 h, then incubated overnight at 4°C with specific primary antibodies. After washing with PBST, membranes were incubated with HRP-conjugated secondary antibodies for 1 h at room temperature. Protein bands were visualized using chemiluminescent substrate and detected using a Tanon 5200 Chemiluminescence Imaging System (Tanon, China).

Transcriptomics Profiling: HRMECs were transfected with CHKA siRNA or scramble siRNA for 24 h. Total RNAs were isolated from each group using a standard extraction kit. RNA purity and concentration were assessed using a NanoDrop 2000 Spectrophotometer (Thermo Fisher Scientific, USA). RNA integrity was verified with an Agilent 2100 Bioanalyzer and the RNA Nano 6000 Assay Kit (Agilent Technologies, USA). After quality control, cDNA libraries were prepared and sequenced using the Illumina platform. The raw sequencing reads underwent quality control and were then aligned to the reference genome. Transcript abundance was calculated using fragments per kilobase of transcript per million mapped reads (FPKM). Differentially expressed genes (DEGs) were identified, and subsequent GO enrichment analysis and Gene Set Enrichment Analysis (GSEA) were performed.

Untargeted Metabolomics Profiling: Untargeted metabolomics profiling was conducted using liquid chromatography-mass spectrometry (LC-MS) on HRMECs transfected with CHKA siRNA or scramble siRNA. Metabolites were extracted by adding 400 μL of extraction solvent (methanol: acetonitrile: water = 2:2:1, v/v). The cell suspension underwent three freeze-thaw cycles and was sonicated on ice for 10 min, followed by centrifugation at 12,000 rpm for 15 min at 4°C. The supernatant was collected for subsequent analysis. Chromatographic separation was conducted on a Waters ACQUITY UPLC BEH Amide column (2.1 mm × 100 mm, 1.7 μm) using a Vanquish UHPLC system (Thermo Fisher Scientific, USA), coupled with a Thermo Q Exactive HFX mass spectrometer for both primary and secondary MS data acquisition. Metabolites were identified through the mzCloud, mzVault, and MassList databases. Only metabolites with a coefficient of variance below 30% in QC samples were considered for analysis. Metabolomic data processing, including peak recognition and integration, was performed using an R-based package with an XCMS kernel. Orthogonal Partial Least Squares Discriminant Analysis (OPLS-DA) was applied to identify metabolic differences between groups.

Mendelian Randomization (MR) Analysis: For MR analysis, we investigated the causal effect of CHKA expression on DR risk using genome-wide association study (GWAS) summary data. CHKA expression quantitative trait loci (eQTL) data were obtained from the IEU OpenGWAS database (dataset eqtl-a-ENSG00000110721), while GWAS summary statistics for DR were obtained from the UK Biobank (UKB) database. We used the “TwoSampleMR” R package to evaluate the causal relationship

between CHKA (exposure) and DR (outcome). Instrumental variables (single nucleotide polymorphisms, SNPs) associated with CHKA expression were selected using the “extract_instruments” function. Data harmonization was performed using the “harmonise_data” function to align effect alleles. SNPs with pleiotropic effects or those strongly related to the outcome were excluded to reduce confounding. Causal estimates were calculated using five MR methods: inverse variance weighted (IVW), MR Egger, weighted median, simple mode, and weighted mode. A causal relationship was considered statistically significant at $P < 0.05$. Results were visualized using scatter plots to illustrate potential causal effects.

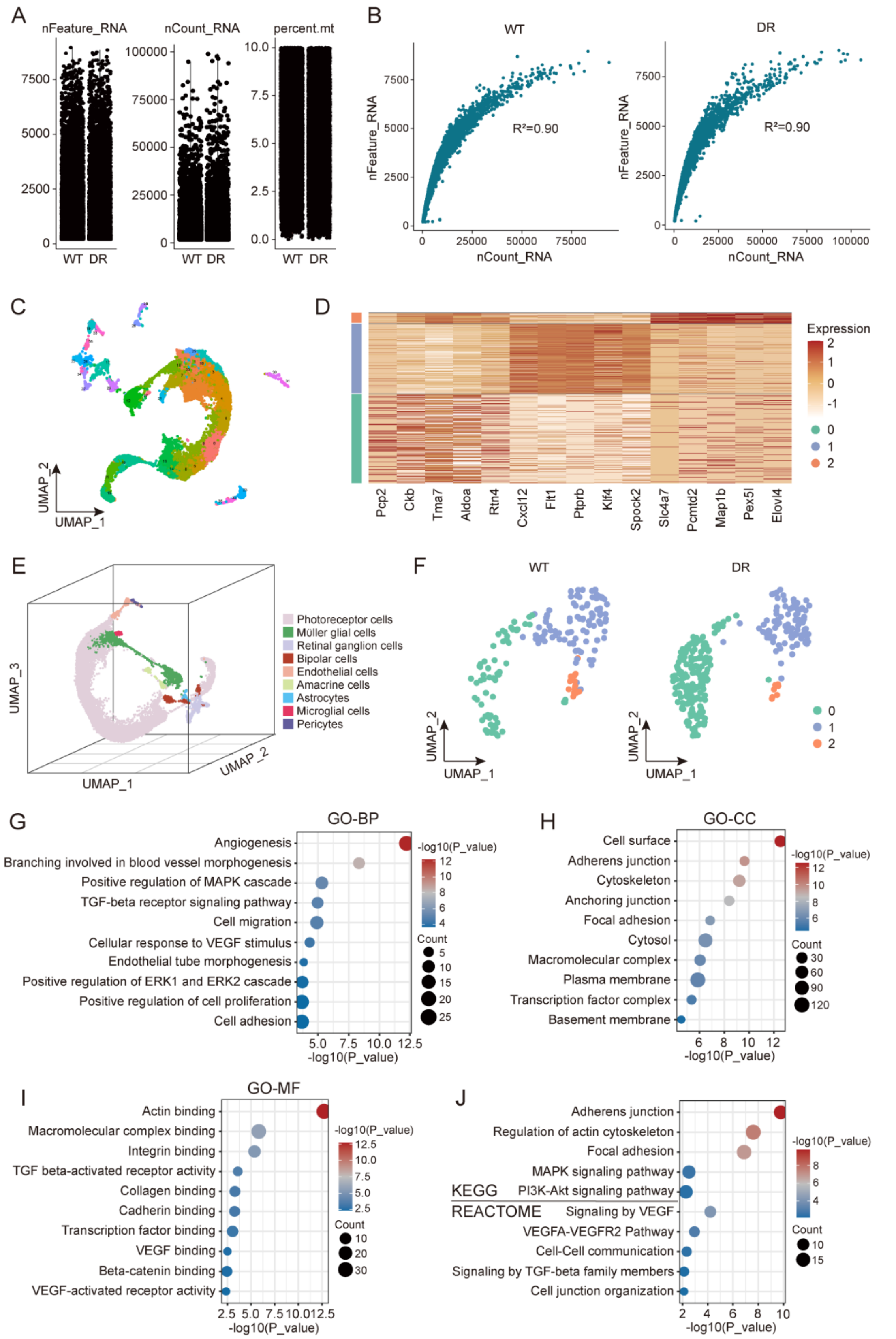

Figure S1. Single-cell analysis reveals distinct transcriptional profiles and functional insights of endothelial cell subclusters

(A) The quality control criteria for each sequenced cell are illustrated, including the number of genes in each cell (nFeature_RNA), the total count of all genes in each cell (nCount_RNA), and the percentage of mitochondrial genes in the total gene count in each cell (percent.mt). (B) A Pearson's correlation analysis was conducted to examine the correlations between nFeature_RNA and nCount_RNA using the Seurat package. (C) UMAP plot displays distinct cell clusters. Each color corresponds to a specific cluster. (D) Heatmap displays the expression profiles of five highly expressed genes across each endothelial cell subcluster. (E) 3D UMAP plot showing 9 cell types. (F) UMAP plots showing three subclusters of endothelial cells in the WT and DR groups. (G-I) Dot plots illustrate the Gene Ontology (GO) enrichment analysis for the top 300 highly expressed genes in endothelial cell subcluster 1, including three categories: biological process (BP, G), cellular component (CC, H), and molecular function (MF, I). (J) Dot plot shows the pathway enrichment results derived from two independent databases, KEGG and REACTOME, using the top 300 highly expressed genes in endothelial cell subcluster 1 as inputs.

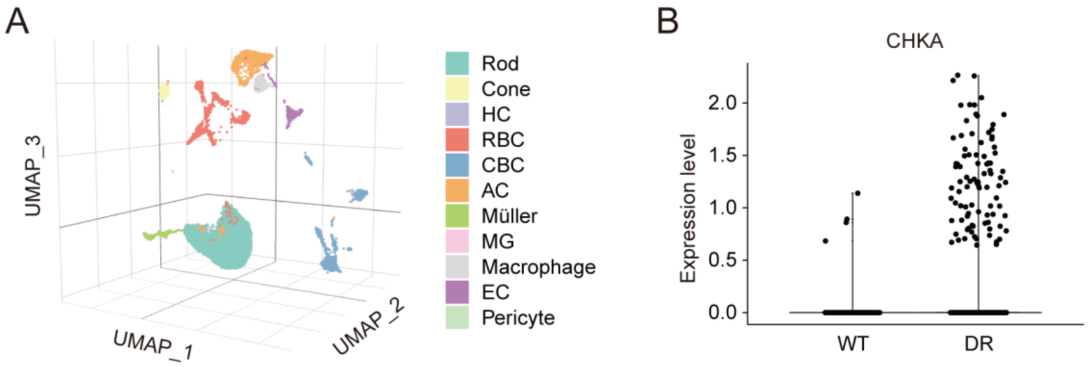

Figure S2. Cross-species validation of CHKA up-regulation in retinal endothelial cells from diabetic rat model

(A) 3D UMAP projection of retinal cell populations, colored by cell types. HC, horizontal cell; RBC, rod bipolar cell; CBC, cone bipolar cell; AC, amacrine cell; Müller, Müller glial cell; MG, microglial cell; EC, endothelial cell. (B) Violin plot showing CHKA expression in retinal endothelial cells from WT and DR groups. CHKA is significantly up-regulated in DR group.

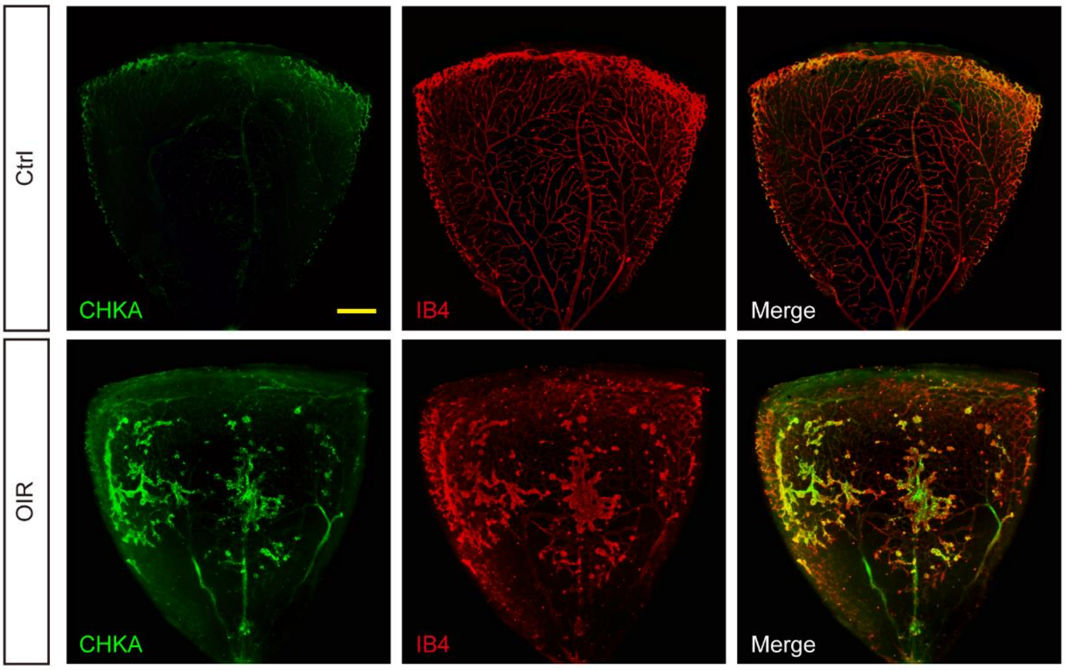

Figure S3. CHKA is involved in pathological retinal neovascularization in OIR model

Representative images of retinal flat mounts demonstrating the co-localization between retinal neovascular tufts and CHKA in OIR retina (n = 5). IB4 staining was conducted to label retinal vessels. Scale bar: 100 μm.

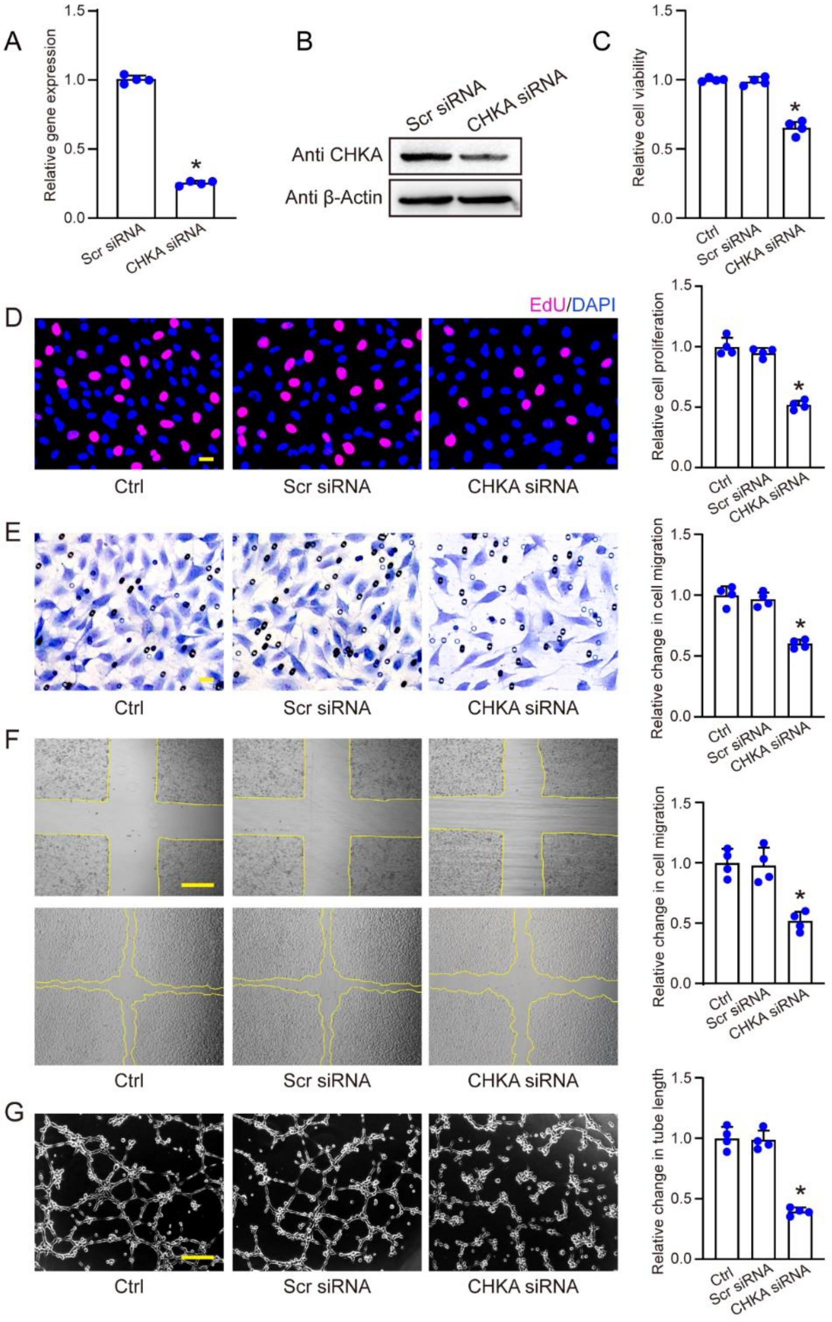

Figure S4. CHKA knockdown exerts anti-angiogenic effects in endothelial cells
HRMECs were transfected with scramble (Scr) siRNA, CHKA siRNA, or left untreated as the control (Ctrl). (A) CHKA gene expression levels were assessed by qRT-PCRs 24 h post-transfection. n = 4; $*P < 0.05$; Student's *t* test. (B) Protein expression levels of CHKA were evaluated 48 h post-transfection through western blots. (C) Cell viability was detected by CCK-8 assay. (D) Cell proliferation was detected by EdU staining. Cell migration was detected by Transwell migration assay (E) and wound healing assay (). (G) Tube formation ability of HRMECs was assessed. n = 4; $*P < 0.05$; One-way ANOVA with Bonferroni post hoc test.

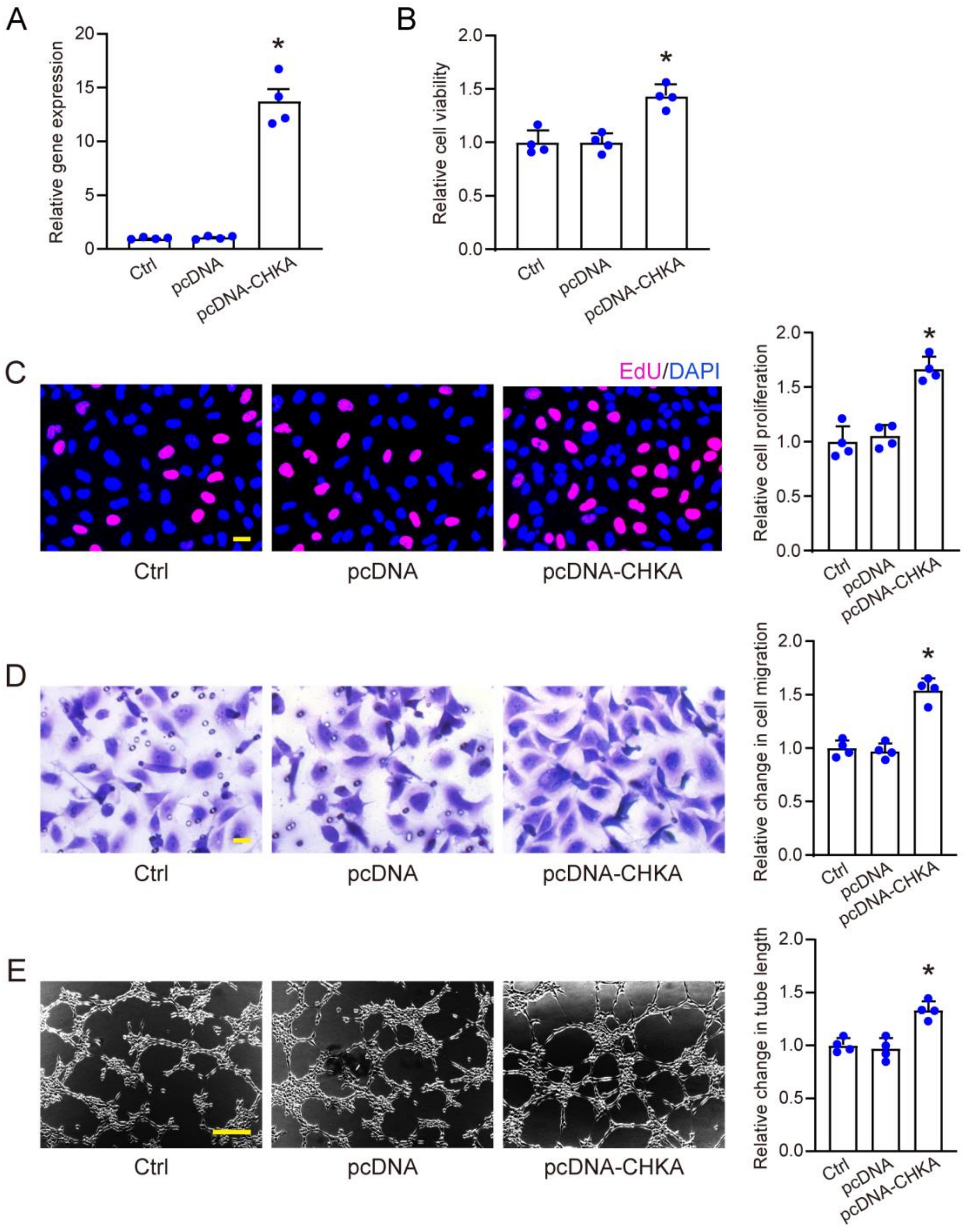

Figure S5. CHKA overexpression enhanced endothelial angiogenic ability *in vitro*
(A) HRMECs were transfected with vector plasmid (pcDNA), CHKA overexpression plasmid (pcDNA-CHKA), or left untreated (Ctrl) for 24 h. The levels of CHKA expression were detected by qRT-PCRs. n = 4; **P* < 0.05; Student's *t* test. (B) Cell viability was detected by CCK-8 assay. (C) Cell proliferation was detected by EdU staining. (D) Transwell assay was conducted to detect cell migration ability. (E) Tube formation ability of HRMECs was detected. = 4; **P* < 0.05; One-way ANOVA with Bonferroni post hoc test.

A

Exposure	Outcome	Method	nsnp	pval	OR (95% CI)	pleio_P
CHKA	Diabetic retinopathy || DM_RETINOPAT_NOS	MR Egger	4	0.960	1.000 (0.997 to 1.003)	0.508
CHKA	Diabetic retinopathy || DM_RETINOPAT_NOS	Weighted median	4	0.017	1.001 (1.000 to 1.002)	0.508
CHKA	Diabetic retinopathy || DM_RETINOPAT_NOS	IVW	4	0.009	1.001 (1.000 to 1.002)	0.508
CHKA	Diabetic retinopathy || DM_RETINOPAT_NOS	Simple mode	4	0.129	1.002 (1.000 to 1.003)	0.508
CHKA	Diabetic retinopathy || DM_RETINOPAT_NOS	Weighted mode	4	0.099	1.001 (1.000 to 1.003)	0.508

B

Outcome	Exposure	Method	Q	Q_df	Q_pval
Diabetic retinopathy || DM_RETINOPAT_NOS	CHKA	MR Egger	0.303	2	0.859
		Inverse variance weighted	0.941	3	0.815

C

Outcome	Exposure	egger_intercept	se	pval
Diabetic retinopathy || DM_RETINOPAT_NOS	CHKA	0.0001	0.0001	0.5081

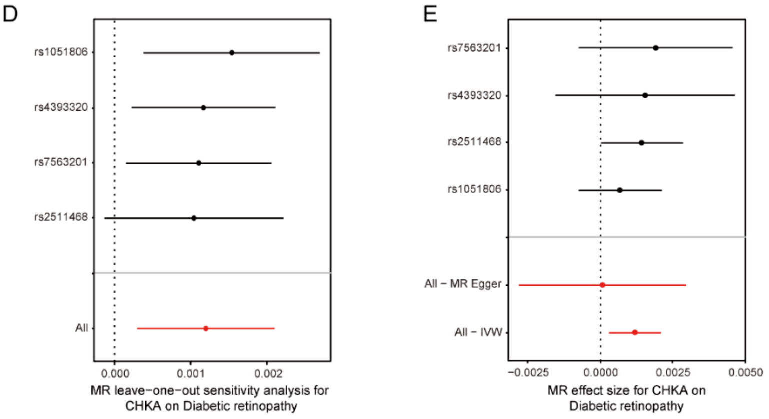

Figure S6. Mendelian Randomization (MR) analysis of CHKA in DR

(A) The results of MR analyses using different statistical methods: MR Egger, Weighted median, Inverse-Variance Weighted (IVW), Simple mode, and Weighted mode. The exposure is CHKA, and the outcome is DR (DM_RETINOPAT_NOS). The number of SNPs used in the analysis (nsnp) is 4. The *p*-values (pval) indicate the statistical significance of the association, with significant results highlighted in red ($P < 0.05$). (B) The heterogeneity of MR results was assessed using Cochran's Q statistic. The Q values, degrees of freedom (Q_df), and p-values (Q_pval) are reported for MR Egger and IVW methods. Non-significant Q_pval ($P > 0.05$) indicate no evidence of heterogeneity. (C) The Egger intercept test was used to assess horizontal pleiotropy in MR analysis. The intercept, standard error (SE), and p-value are reported. Non-significant p-values ($P > 0.05$) indicate no evidence of pleiotropy. (D) Leave-one-out sensitivity analysis for CHKA on DR. The red point represents the combined effect estimate including all SNPs, while the black points represent estimates excluding one SNP at a time. The dashed line indicates the null effect. (E) Forest plot of MR effect sizes for CHKA on DR by MR Egger and IVW methods. The black points represent the effect sizes for individual SNPs, and the red points represent the combined estimates.
